# Knockout of Targeted Plasmid-Borne β-Lactamase Genes in an Extended-Spectrum-β-Lactamase-Producing Escherichia coli Strain: Impact on Resistance and Proteomic Profile

**DOI:** 10.1128/spectrum.03867-22

**Published:** 2023-01-09

**Authors:** Daniel Jaén-Luchoro, Roger Karlsson, Antonio Busquets, Beatriz Piñeiro-Iglesias, Nahid Karami, Nachiket P. Marathe, Edward R. B. Moore

**Affiliations:** a Department of Infectious Diseases, Institute for Biomedicine, Sahlgrenska Academy of the University of Gothenburg, Gothenburg, Sweden; b Centre for Antibiotic Resistance Research, University of Gothenburg, Gothenburg, Sweden; c Culture Collection University of Gothenburg, Department of Clinical Microbiology, Sahlgrenska University Hospital, Region Västra Götaland and Sahlgrenska Academy, University of Gothenburg, Gothenburg, Sweden; d Department of Clinical Microbiology, Sahlgrenska University Hospital, Region Västra Götaland, Gothenburg, Sweden; e Nanoxis Consulting AB, Gothenburg, Sweden; f Microbiology, Department of Biology, University of the Balearic Islands, Palma de Mallorca, Spain; g Institute of Marine Research, Bergen, Norway; Brown University

**Keywords:** β-lactamase, ESBL, *Escherichia coli*, quantitative proteomics, tandem mass tag

## Abstract

Resistance to β-lactams is known to be multifactorial, although the underlying mechanisms are not well established. The aim of our study was to develop a system for assessing the phenotypic and proteomic responses of bacteria to antibiotic stress as a result of the loss of selected antimicrobial resistance genes. We applied homologous recombination to knock out plasmid-borne β-lactamase genes (*bla*_OXA-1_, *bla*_TEM-1_, and *bla*_CTX-M15_) in Escherichia coli CCUG 73778, generating knockout clone variants lacking the respective deleted β-lactamases. Quantitative proteomic analyses were performed on the knockout variants and the wild-type strain, using bottom-up liquid chromatography tandem mass spectrometry (LC-MS/MS), after exposure to different concentrations of cefadroxil. Loss of the *bla*_CTX-M-15_ gene had the greatest impact on the resulting protein expression dynamics, while losses of *bla*_OXA-1_ and *bla*_TEM-1_ affected fewer proteins’ expression levels. Proteins involved in antibiotic resistance, cell membrane integrity, stress, and gene expression and unknown function proteins exhibited differential expression. The present study provides a framework for studying protein expression in response to antibiotic exposure and identifying the genomic, proteomic, and phenotypic impacts of resistance gene loss.

**IMPORTANCE** The critical situation regarding antibiotic resistance requires a more in-depth effort for understanding underlying mechanisms involved in antibiotic resistance, beyond just detecting resistance genes. The methodology presented in this work provides a framework for knocking out selected resistance factors, to study the adjustments of the bacterium in response to a particular antibiotic stress, elucidating the genetic response and proteins that are mobilized. The protocol uses MS-based determination of the proteins that are expressed in response to an antibiotic, enabling the selection of strong candidates representing putative resistance factors or mechanisms and providing a basis for future studies to understand their implications in antibiotic resistance. This allows us to better understand how the cell responds to the presence of the antibiotic when a specific gene is lost and, consequently, identify alternative targets for possible future treatment development.

## INTRODUCTION

Gram-negative bacteria, including members of the *Enterobacteriaceae*, such as extended-spectrum-β-lactamase (ESBL)-producing enteric bacteria, are particularly problematic (1, 2). The World Health Organization (WHO) has included ESBL-producing *Enterobacteriaceae* in the WHO Global Priority Pathogens List as “priority 1: critical” (3), and the Centers for Disease Control and Prevention (CDC, USA) has classified ESBL-producing bacteria in the category of “serious threat,” based on clinical and economic impact, incidence, 10-year projection of incidence, transmissibility, availability of effective antibiotics, and barriers to prevention (2).

Extensive efforts are necessary to better understand resistance mechanisms, beyond the three canonical classes accepted to date, i.e., target modification, drug inactivation, and drug transport (4, 5). Although expression of genes directly responsible for such mechanisms is essential for phenotypic resistance, other genetic factors that may be involved directly or indirectly in controlling expression or complementing function are largely unknown (6). Combining genomics and proteomics approaches facilitates comprehensive studies, wherein the proteomic responses of genes can be determined in their genomic and phenotypic contexts, defining the extent to which the genes are expressed under specific conditions (7, 8). Proteomics-based studies have been carried out to better understand the overall metabolic changes and sets of proteins or pathways that are involved in the response to antibiotic stress (9–12). These studies have shown that diverse bacteria respond differently to a given antibiotic and that β-lactam resistance is a multifactorial response that involves membrane modifications, DNA/RNA modifications, energy and central carbon metabolism, and even chemotaxis (6, 13, 14). With the existing evidence, the significance and relationships of the underlying mechanisms are poorly understood and unexplored, particularly in multiresistant pathogens (6). These kinds of genomic-proteomic studies can lead to a better understanding of the response of a bacterium to an antibiotic or combination of antibiotics, as well as helping to identify important proteins or pathways that may represent targets for the development of new antibiotics. However, standard methods for this kind of analysis, including studying the effect of resistance gene loss on the proteome, are largely lacking.

The aim of this study was to design an efficient protocol for targeting and deleting, or knocking out, specific plasmid-encoded β-lactamase genes and for observing the impacts that the knockout of specific resistance genes has on the overall protein expression dynamics and the phenotypic resistance of the bacterium. Gene deletion was performed using homologous recombination, and a quantitative global proteomics analysis was performed with tandem mass tags (TMT) with bottom-up, liquid chromatography-tandem mass spectrometry (LC-MS/MS) for determining differentially expressed proteins.

## RESULTS AND DISCUSSION

Proteomic studies have been carried out previously on bacterial strains with known resistance genes, in order to identify proteins or pathways that are impacted by the presence of a specific antibiotic (9–12). The approach of these studies leads to recognition of the mechanisms that are activated or repressed when antibiotic-resistant bacteria encounter antibiotic stress conditions, i.e., beyond the particular resistance gene. In fact, evidence has shown that some clinical isolates and strains exhibit high levels of resistance to β-lactams, mainly due to decreased uptake capacity or increased efflux, rather than the β-lactamase itself (15). Such observations indicate that the resistance genes are not the only genetic factors involved in generating phenotypic resistance.

To the best of our knowledge, this is the first study reporting a protocol for the targeted deletion, or knockout, of selected plasmid-encoded antibiotic resistance genes in a multiresistant ESBL-producing Escherichia coli strain (wild-type [WT] E. coli), CCUG 73778, and the assessment of the phenotypic impact and the effects on the global protein network produced by each gene loss, in response to differing antibiotic stress conditions in clinical E. coli isolates.

### Confirmation of knockout clone variants.

The gene knockout protocol generated three different clone variants of E. coli CCUG 73778 (E. coli WT): one lacking the ESBL *bla*_CTX-M-15_ (E. coli ΔCTX, referred to here as ΔCTX), one lacking the *bla*_OXA-1_ β-lactamase gene (E. coli ΔOXA, referred to here as ΔOXA), and another lacking the *bla*_TEM-1_ β-lactamase gene (E. coli ΔTEM, referred to here as ΔTEM). The clones of each variant were confirmed by PCR amplification and Sanger sequencing. Final confirmation was performed with whole-genome sequencing of the clone variants, using Illumina NovaSeq.

Mapping of the Illumina reads of each knockout clone variant was performed against the complete genome sequence of E. coli CCUG 73778 (accession numbers CP041337 to CP041343). Figure S1 in the supplemental material shows the section of E. coli CCUG 73778 plasmid pSUH-1 where *bla*_TEM-1_ is located (Fig. S1A) and the section of plasmid pSUH-2 (Fig. S1B) where *bla*_OXA-1_ and *bla*_CTX-M-15_ are located. Both are IncF plasmids, a family of plasmids highly prevalent in clinics and capable of being propagated among members of the order *Enterobacterales*. In each clone variant, only the targeted gene was selectively removed.

### Antibiotic sensitivity testing.

The overall resistance-sensitivity profiles (Table 1) show that the knockout clone variants ΔOXA and ΔTEM exhibited the same resistance profile as the E. coli WT. The major change in the overall phenotypic resistance profile was observed in the knockout clone ΔCTX. In this case, as the main defense against cephalosporins had been eliminated, the clone variant was sensitive to all cephalosporins, except for cefuroxime, to which it appears to be intermediately resistant. The MIC of cefadroxil was included after disc diffusion tests had been performed (Table S1). Since the most dramatic change in the phenotypic resistance profile was observed in ΔCTX, this clone was used as a guide to select the antibiotic to be used in the present work. As the objective was to understand the response of the strain to cephalosporin exposure following gene loss, the selection of the antibiotic was restricted to this class of antibiotics, specifically to cephalosporins to which ΔCTX was sensitive. In proteome analysis, cefadroxil was the only cephalosporin that allowed adequate growth of ΔCTX at a concentration of 8 μg/mL. Apart from cephalosporins, some variations in MICs also were observed in the different clones for some of the antibiotics (Table 1). It is worth highlighting that, independent of the gene that was knocked out, the MIC of piperacillin-tazobactam was observed to be half that observed for the E. coli WT, in all cases. It has been reported that *bla*_OXA-1_ may contribute to tazobactam resistance (16), which may influence the resistance to this antibiotic in the mutants when this gene is intact. Mutations associated with *bla*_CTX-M-15_ also have been associated with increased resistance to piperacillin-tazobactam (17, 18), although none of these was found in the *bla*_CTX-M-15_ gene in strain CCUG 73778. Overexpression of *bla*_TEM-1_ is another mechanism associated with resistance to piperacillin-tazobactam (19). In the strain CCUG 73778, this gene is preceded by the P4 promoter, which is associated with hyperproduction of this enzyme (20), and this may explain the resistance in the clones in which *bla*_TEM-1_ is still present, particularly in ΔOXA. Additionally, the WT strain and knockout mutants have various β-lactamases, which is a characteristic also linked to resistance against piperacillin-tazobactam (21), among other mechanisms, such as the efflux pump AcrAB (22).

**TABLE 1:** Antibiotic resistance profiles of E. coli CCUG 73778 (E. coli WT) and the three clone variants (ΔOXA, ΔCTX, and ΔTEM)^*a*^

Antibiotic	Class	MIC (μg/mL)			
		E. coli WT	ΔOXA	ΔCTX	ΔTEM
Amikacin	Aminoglycoside	16	16	16	16
Gentamicin	Aminoglycoside	>16	>16	>16	>16
Tobramycin	Aminoglycoside	>16	>16	>16	>16
Trimethoprim	Antifolate	>16	>16	>16	>16
Trimethoprim-sulfamethoxazole	Antifolate	>16	>16	>16	>16
Ertapenem	Carbapenem	0.06	**0.03**	**≤0.015**	0.06
Imipenem	Carbapenem	0.12	0.12	**0.25**	0.12
Meropenem	Carbapenem	0.03	0.03	0.03	0.03
Cefadroxil	Cephalosporin	>16	>16	**16**	>16
Ceftazidime	Cephalosporin	>16	>16	**0.25**	>16
Cephalexin	Cephalosporin	>32	>32	**8**	>32
Ceftriaxone	Cephalosporin	>4	>4	**0.12**	>4
Ceftibuten	Cephalosporin	>4	>4	**0.25**	>4
Cefotaxime	Cephalosporin	>8	>8	**0.25**	>8
Cefuroxime	Cephalosporin	>16	>16	**8**	>16
Cefepime	Cephalosporin	>16	>16	**0.5**	>16
Cefixime	Cephalosporin	>4	>4	**0.5**	>4
Ciprofloxacin	Fluoroquinolone	0.06	0.06	0.06	0.06
Levofloxacin	Fluoroquinolone	≤0.03	≤0.03	≤0.03	**0.06**
Amoxicillin	Penicillin	>32	>32	>32	>32
Amoxicillin-clavulanic acid (fixed 2 mg/L clavulanic acid)	Penicillin	>128	**64**	>128	128
Ampicillin	Penicillin	>32	>32	>32	>32
Piperacillin-tazobactam (fixed 4 mg/L tazobactam)	Penicillin	>64	**32**	**32**	**32**
Temocillin	Penicillin	16	16	**8**	16
Colistin	Polymyxin	0.5	0.5	0.5	0.5
Tigecycline	Tetracycline	0.25	0.25	0.25	0.25
Nitrofurantoin		≤8	≤8	≤8	≤8

a The antibiotic panel used is recommended for E. coli by the National Reference Laboratory for Antibiotic Resistance (Växjö, Sweden), following the EUCAST guidelines. Boldface indicates differences in MIC with respect to the WT.

In order to further study the effects of the loss of each gene on the proteome, the knockout clone variants and the E. coli WT were grown in the presence of cefadroxil. The maximum concentration allowing sufficient growth was 8 μg/mL. With this concentration as a reference, three concentration points were established: 0 μg/mL as the starting point, 4 μg/mL as the medium inhibitory concentration (MID), and 8 μg/mL as the subMIC.

### Proteomic analysis.

**FIG 1:**
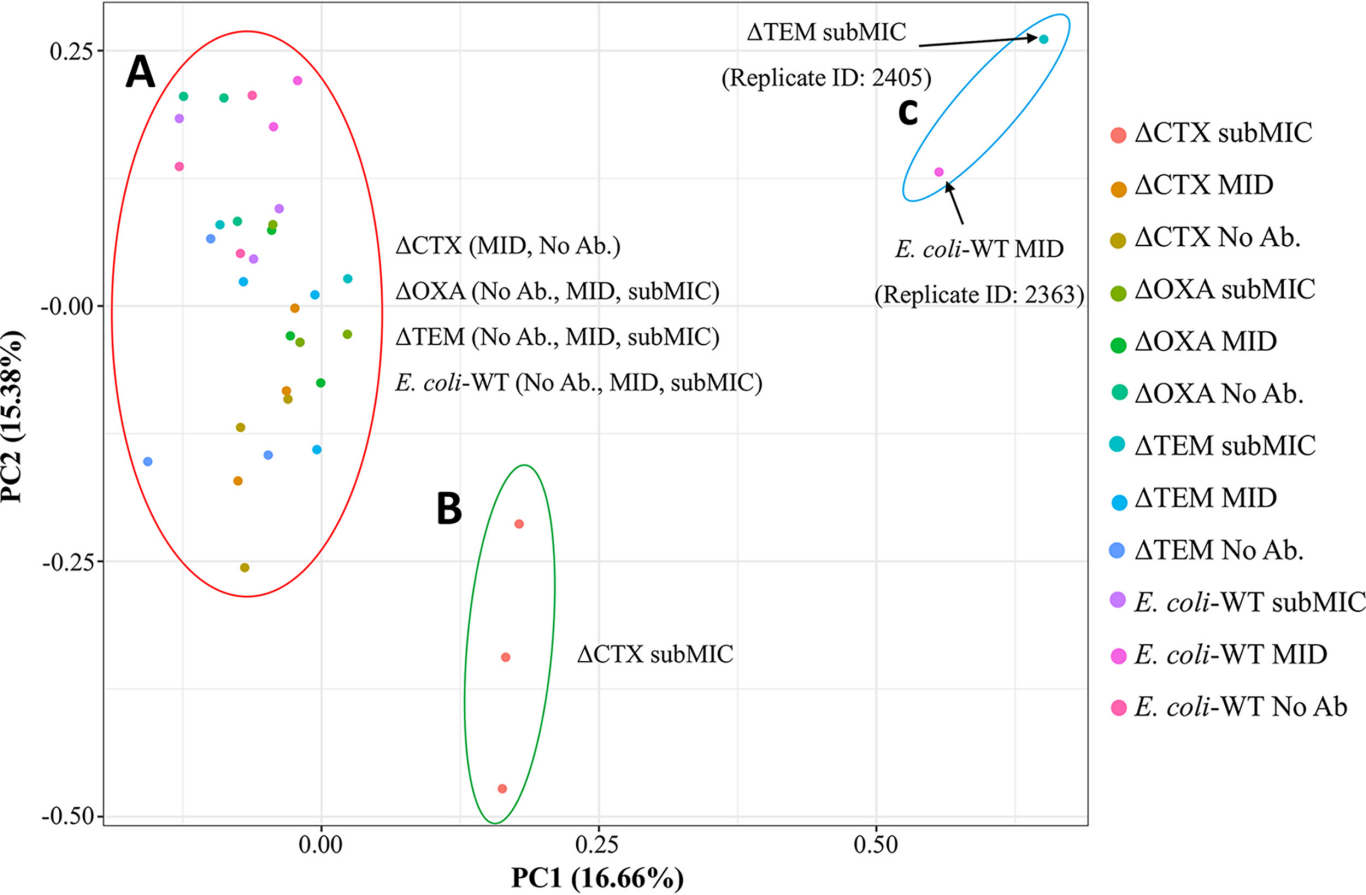
PCA plot based on the proteomic analysis results. Results were obtained by LC-MS/MS of the triplicate samples exposed to three concentrations of cefadroxil (no antibiotic [No Ab.], 4 μg/mL [MID], and 8 μg/mL [subMIC]) for the E. coli WT and the three knockout clone variants. The plot shows three groups of samples, according to their similarity by PC1 and PC2: group A, with representatives of all samples, except the triplicate samples of ΔCTX exposed to subMIC of antibiotic (group B), and group C, which shows the two outliers found.

After the samples were analyzed by quantitative, bottom-up, LC-MS/MS-based shotgun proteomics, the results of identified peptides were analyzed by principal-component analysis (PCA), to determine the distribution of samples. PCA plots showed three clear groups of samples (Fig. 1). The first group includes representatives of all samples, except ΔCTX exposed to the subMIC of cefadroxil, which forms a distinct group separated from the rest (Fig. 1A and B). Additionally, one replicate from ΔTEM exposed to the subMIC and one replicate of the E. coli WT exposed to the MID were positioned away from the rest (Fig. 1C). These two samples are separated by PC1 from the other two replicates of the same sample in each case, which are included in the first group described. As they were acting as outliers, they were not considered in downstream analysis. The overall distribution seen in the PCA plot indicated that ΔCTX at the subMIC was the most different sample from the rest. The hypothesis for this PCA plot distribution would be that the WT has all defense mechanisms against the antibiotic intact, which allows the strain to face the presence of the antibiotic with basal or small modifications of the expression of β-lactamases and minimal adjustments in the proteome. Clones with intact *bla*_CTX-M15_ have the main defense against cephalosporins still present and, hence, are able to respond similarly to the WT, at least at first consideration. However, clone ΔCTX is missing this β-lactamase, which causes it to express more intensive changes as the antibiotic concentration increases, especially at the maximal concentration used.

**FIG 2:**
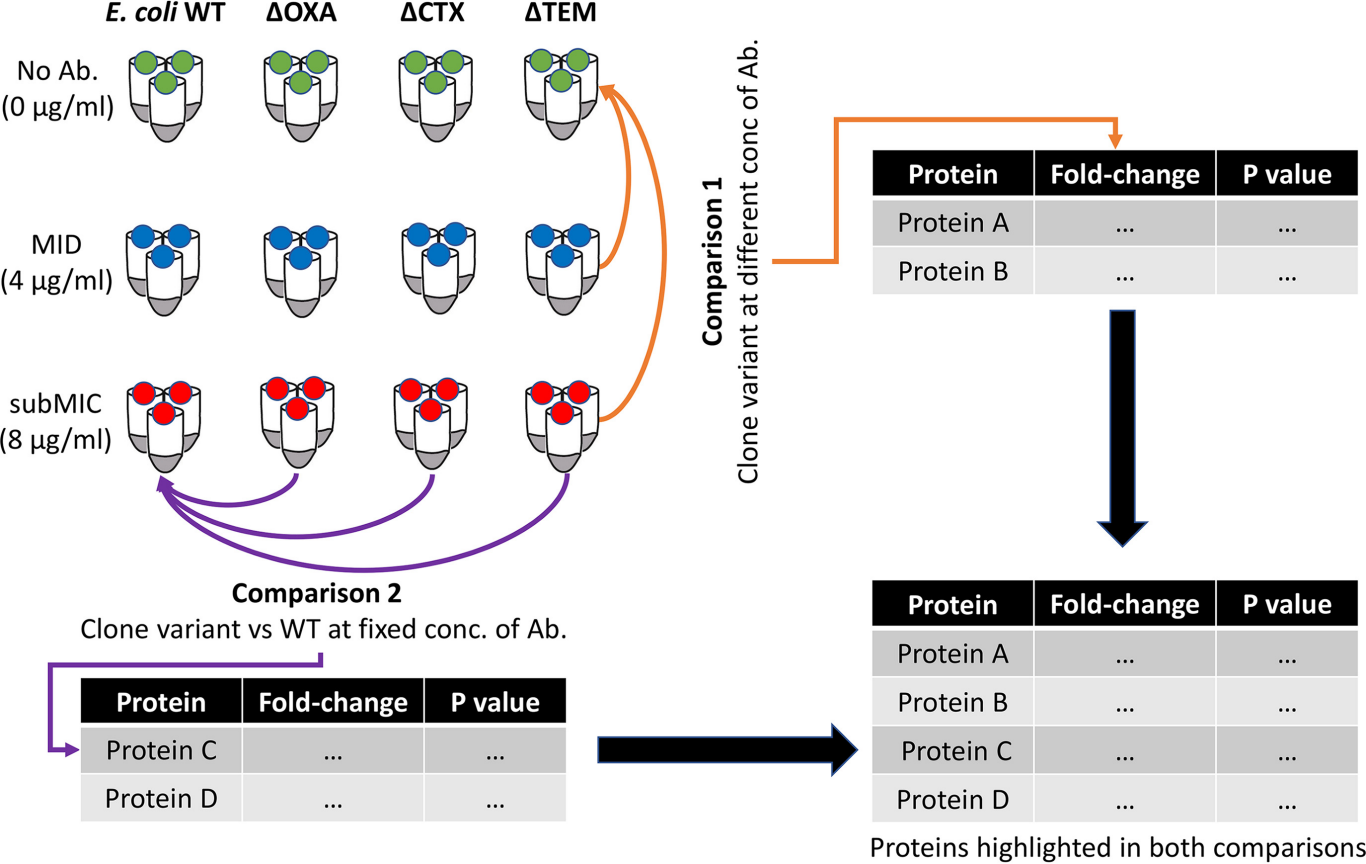
Schematic showing the experimental design of the two comparisons of protein expression performed. In comparison 1, the list of proteins resulted from comparing each knockout clone variant exposed to different concentrations of antibiotics to the same clone variant with no exposure to antibiotics (MID over no antibiotic [Ab.] and subMIC over no antibiotic). Comparison 2 resulted from the comparison of each knockout clone variant to the E. coli WT at fixed concentrations of antibiotic (no antibiotic over no antibiotic, MID over MID, and subMIC over subMIC). The final list of proteins summarized the proteins highlighted in both comparisons.

The results obtained by proteomic analysis for the three different conditions (i.e., exposure to different concentrations of the selected antibiotic) for each knockout clone variant were compared through two different approaches: comparison 1, where protein expression in a given clone was compared between the different conditions (no antibiotic versus antibiotic at the MID and the subMIC), and comparison 2, where protein expression of each clone variant was compared with that of the E. coli WT strain under the same conditions (no antibiotic, MID, and subMIC) (Fig. 2). The first approach allowed determination of the proteins that varied for each clone when exposed to increasing concentrations of antibiotics in the absence of each knocked-out gene. The second approach allowed the determination of proteins that varied significantly in each knockout compared with the E. coli WT.

### Comparison 1: differential expression of proteins in each clone variant.

In the first set of comparisons, fold changes, that is, ratios of expression levels of a particular proteins in each knockout clone variant compared at two different conditions (i.e., concentration of antibiotic), and Welch’s *t* test *P* values of each comparison were calculated. The conditions “MID versus no antibiotic” and “subMIC versus no antibiotic” were compared separately for the E. coli WT and for each clone variant (Fig. 2), to determine differential protein expression, after the removal of the respective resistance gene, at different antibiotic concentrations. Proteins detected with only one peptide were not considered for further analysis. Volcano plots were created, based on fold changes and Welch’s *t* test *P* values, to determine the overall pictures of the protein expression profile in each strain (Fig. S2). The volcano plots showed that the E. coli WT and ΔTEM have only one protein passing the applied thresholds and statistically significant cutoff (fold changes of ≥1.5 or ≤−1.5; *P* value, ≤0.05), regardless of the concentration of antibiotics applied (MID or subMIC). On the other hand, ΔOXA exhibited similar numbers of proteins differentially expressed under MID and subMIC conditions, whereas ΔCTX had a significantly higher number of proteins differentially expressed at the subMIC of antibiotic.

The numbers of proteins with differential expression in each knockout clone variant and the E. coli WT, after filtering the results obtained by using specific thresholds, are presented in Table 2; annotation of all identified proteins is listed in Appendix S1. Only one overexpressed protein was listed for the E. coli WT (biotin synthase) and ΔTEM (cytochrome *o* ubiquinol oxidase subunit I). ΔOXA seemed to be more affected, but with similar numbers of proteins passing the thresholds under MID and subMIC conditions (25 and 21 proteins in total, respectively). Finally, ΔCTX showed a total of 58 proteins passing the thresholds, of which 23 are overexpressed and 35 present a lower expression under subMIC conditions, even though only 3 proteins were highlighted under MID conditions.

**TABLE 2:** Numbers of proteins with differential expression in each clone under MID and subMIC conditions, determined in comparison 1

Strain	No. of proteins			
	MID vs no antibiotic		subMIC vs no antibiotic	
	Higher expression	Lower expression	Higher expression	Lower expression
WT	0	0	1	0
ΔOXA	22	3	20	1
ΔCTX	2	1	23	35
ΔTEM	0	0	1	0

The specific details of proteins highlighted for the ΔOXA clone variant are listed in Table 3. Most proteins showed significant changes in both MID and subMIC conditions, with respect to the same knockout clone variant not exposed to antibiotics. Additionally, some proteins showed a significant fold change but not statistically significant *P* value at the MID concentration, although they clearly exhibited a significant fold change and *P* value at the subMIC. Hence, when the data were evaluated, the proteins passing the specific thresholds under the subMIC conditions were used, whereas the MID condition was used to observe the trend of expression of those proteins.

**TABLE 3:** Proteins demonstrating higher or lower expression in a comparison of ΔOXA treated with MID and subMIC antibiotic to ΔOXA not exposed to antibiotic^*a*^

Accession no.^*b*^	Description	FC for MID^*c*^	*P* value^*d*^	FC for subMIC^*c*^	*P* value^*d*^
QGA32136	Major outer membrane lipoprotein	3.97	0.02	3.33	0.02
QGA33873	Molecular chaperone OsmY	2.92	0.02	2.78	0.02
QGA34791	Acid-activated periplasmic chaperone HdeB	2.33	0.04	2.28	0.04
QGA32166	Superoxide dismutase [Cu-Zn] SodC2	2.44	0.00	2.19	0.01
QGA32269	Stationary-phase-induced ribosome-associated protein	2.17	0.02	2.15	0.03
QGA31305	Phosphocarrier protein Hpr	2.39	0.02	2.14	0.03
QGA33473	Phosphate starvation-inducible protein PsiF	2.22	0.04	1.93	0.05
QGA32890	DUF2190 family protein	1.72	0.05	1.86	0.04
QGA33651	Rho-binding antiterminator	2.19	0.03	1.81	0.05
QGA32001	Transcription antiterminator/RNA stability regulator CspE	1.86	0.04	1.76	0.05
QGA34355	PTS fructose-like transporter subunit IIB	1.57	0.04	1.75	0.02
QGA34143	Cochaperone GroES	1.67	0.04	1.70	0.04
QGA33038	Heavy metal-binding domain-containing protein	1.65	0.03	1.70	0.02
QGA33628	C-lysozyme inhibitor	1.67	0.04	1.68	0.04
QGA34857	DUF2756 family protein	1.91	0.05	1.68	0.05
QGA35156	Bifunctional threonine ammonia-lyase/l-serine ammonia-lyase TdcB	1.47	0.07	1.65	0.03
QGA32706	50S ribosomal protein L32	1.74	0.06	1.63	0.01
QGA35701	Potassium binding protein Kbp	1.38	0.09	1.57	0.05
QGA31167	Autonomous glycyl radical cofactor GrcA	1.48	0.04	1.55	0.03
QGA32296	Aminobutyraldehyde dehydrogenase	−1.09	0.15	1.53	0.00
QGA31363	Long-chain fatty acid transporter FadL	−2.45	0.03	−2.26	0.04

a The results are sorted by proteins demonstrating significantly differential expression in ΔOXA at subMIC compared with no antibiotic exposure.

b NCBI protein accession numbers.

c Protein FCs ≥1.5 and ≤−1.5 at subMIC concentration are shown.

d Calculated with Welch’s *t* test.

In the case of ΔCTX, the list of differentially expressed proteins was much longer than that for the other clone variants when filtered by the results under subMIC conditions (Table 4). Finally, in the case of ΔTEM, since only one protein was statistically differentially expressed (Appendix S1), no further comparisons were carried out.

**TABLE 4:** Proteins demonstrating higher or lower expression in a comparison of ΔCTX treated with MID and subMIC antibiotic to ΔCTX not exposed to antibiotic^*a*^

Accession no.^*b*^	Description^*c*^	FC for MID^*d*^	*P* value^*e*^	FC for subMIC^*d*^	*P* value^*e*^
QGA31512	DUF2303 family protein	2.69	0.00	5.37	0.00
QGA36195	Oxacillin-hydrolyzing class D beta-lactamase OXA-1 (plasmid)	1.13	0.33	2.28	0.01
QGA35118	DEAD/DEAH family ATP-dependent RNA helicase	1.24	0.15	2.24	0.00
QGA34611	Heat shock chaperone IbpB	1.39	0.02	2.21	0.00
QGA35331	KpsF/GutQ family sugar-phosphate isomerase	−1.05	0.76	2.09	0.01
QGA31547	Phage tail protein	1.50	0.03	2.03	0.01
QGA33125	Biotin synthase	−1.19	0.49	1.93	0.04
QGA32840	l,d-Transpeptidase	1.12	0.06	1.76	0.00
QGA33763	Cell division protein FtsL	1.10	0.62	1.72	0.04
QGA33114	Bax inhibitor-1/YccA family protein	1.12	0.05	1.71	0.00
QGA33718	Glucose/quinate/shikimate family membrane-bound PQQ-dependent dehydrogenase	1.10	0.05	1.66	0.02
QGA36007	Class A broad-spectrum beta-lactamase TEM-1 (plasmid)	1.12	0.02	1.62	0.02
QGA33094	Mechanosensitive channel protein	1.08	0.50	1.59	0.01
QGA34675	3-Deoxy-d-manno-oct-2-ulosonate III transferase WaaZ	−1.04	0.83	1.59	0.04
QGA36177	Replication regulatory protein RepA (plasmid)	1.07	0.65	1.56	0.05
QGA35694	DUF883 domain-containing protein	1.08	0.12	1.56	0.00
QGA34782	Glutamate decarboxylase	1.15	0.03	1.54	0.00
QGA34892	RNA-binding transcriptional accessory protein	1.00	0.92	1.53	0.00
QGA34181	Glycine betaine/l-proline transporter ProP	1.04	0.56	1.52	0.01
QGA33887	Phosphatidylglycerol–membrane-oligosaccharide glycerophosphotransferase	1.11	0.18	1.51	0.00
QGA33090	DNA starvation/stationary-phase protection protein	1.16	0.02	1.50	0.01
QGA31459	Anaerobic glycerol-3-phosphate dehydrogenase subunit C	1.09	0.22	1.50	0.01
QGA35923	YgdI/YgdR family lipoprotein	1.10	0.31	1.50	0.01
QGA36126	Conjugal transfer protein TraB (plasmid)	−1.21	0.15	−1.50	0.02
QGA32192	TetR/AcrR family transcriptional regulator	−1.04	0.53	−1.50	0.00
QGA32412	Tryptophan synthase subunit beta	−1.03	0.54	−1.51	0.00
QGA32413	Tryptophan synthase subunit alpha	1.08	0.44	−1.51	0.04
QGA32411	Bifunctional indole-3-glycerol-phosphate synthase TrpC/phosphoribosylanthranilate isomerase TrpF	−1.01	0.99	−1.52	0.01
QGA35498	Xanthine dehydrogenase molybdenum-binding subunit XdhA	−1.02	0.65	−1.54	0.00
QGA32197	Class II fumarate hydratase	1.01	0.88	−1.54	0.01
QGA31396	Histidine ABC transporter substrate-binding protein HisJ	−1.03	0.76	−1.54	0.01
QGA34311	Two-component system sensor histidine kinase ZraS	−1.07	0.14	−1.55	0.00
QGA35486	Putative aminohydrolase SsnA	1.03	0.56	−1.57	0.01
QGA35484	Molybdopterin-dependent oxidoreductase Mo/Fe-S-binding subunit	1.08	0.05	−1.59	0.00
QGA34751	Kdo(2)-lipid A phosphoethanolamine 7′′-transferase	−1.04	0.53	−1.59	0.00
QGA34718	2,3-Diketo-l-gulonate transporter substrate-binding protein YiaO	−1.10	0.51	−1.59	0.05
QGA35305	DUF4092 domain-containing protein	−1.11	0.03	−1.60	0.01
QGA31399	Histidine ABC transporter ATP-binding protein HisP	−1.05	0.48	−1.60	0.00
QGA35487	Putative selenate reductase subunit YgfK	1.06	0.18	−1.61	0.02
QGA32008	CNNM family cation transport protein YoaE	1.07	0.17	−1.64	0.00
QGA32410	Bifunctional anthranilate synthase glutamate amidotransferase component TrpG/anthranilate phosphoribosyltransferase TrpD	1.12	0.18	−1.64	0.03
QGA35492	d-Phenylhydantoinase	1.07	0.34	−1.66	0.01
QGA35854	DUF2501 domain-containing protein	1.10	0.21	−1.66	0.01
QGA36222	Hypothetical protein FMA85_26960 (plasmid)	−1.03	0.85	−1.69	0.01
QGA35494	Diaminopropionate ammonia-lyase	1.06	0.10	−1.70	0.00
QGA36220	MobC family plasmid mobilization relaxosome protein (plasmid)	−1.10	0.05	−1.70	0.02
QGA32839	DUF882 domain-containing protein	1.13	0.17	−1.70	0.00
QGA35582	MFS transporter	−1.09	0.58	−1.73	0.02
QGA35918	Knotted carbamoyltransferase YgeW	1.17	0.03	−1.73	0.00
QGA32870	DUF3102 domain-containing protein	−1.11	0.04	−1.73	0.05
QGA32871	Helix-turn-helix domain-containing protein	−1.05	0.76	−1.77	0.01
QGA32865	Host-nuclease inhibitor protein Gam	−1.13	0.21	−1.79	0.04
QGA35485	Molybdopterin-dependent oxidoreductase FAD-binding subunit	1.01	0.88	−1.79	0.00
QGA32888	Peptidase	−1.16	0.48	−1.80	0.05
QGA32409	Anthranilate synthase component I	−1.02	0.97	−1.81	0.01
QGA34312	Zinc resistance sensor/chaperone ZraP	−1.15	0.21	−1.82	0.01
QGA33282	Carbon starvation protein CstA	−1.07	0.60	−1.85	0.01
QGA31415	Transcriptional regulator LrhA	−1.12	0.05	−2.01	0.00

a The results are sorted by proteins demonstrating significantly differential expression in ΔCTX at subMIC compared with no antibiotic exposure.

b NCBI protein accession numbers.

c CNNM, CBS-pair domain divalent metal cation transport mediator; FAD, flavin adenine dinecleotide; Kdo, 3-deoxy-d-manno-octulosonic acid; MFS, major facilitator superfamily.

d Protein FCs ≥1.5 and ≤−1.5 at subMIC concentration are shown.

e Calculated with Welch’s *t* test.

### Comparison 2: differentially expressed proteins in each clone in comparison with the E. coli WT at a fixed concentration of antibiotic.

Fold changes and Welch’s *t* test *P* values were calculated, comparing each clone variant with the E. coli WT exposed to the same concentration of antibiotic. Volcano plots based on fold changes and *P* values were created to compare the dispersion of proteins from the no-antibiotic to the subMIC condition (Fig. S3). The numbers of differentially expressed proteins are shown in Table 5, and annotation details of each protein are summarized in Appendix S1.

**TABLE 5:** Numbers of proteins with higher or lower expression determined in comparison 2

Strain compared with the WT	Antibiotic concn	No. of proteins		
		With higher expression	With lower expression	Total
ΔOXA	None	5	13	18
	MID	27	21	48
	subMIC	33	14	47
ΔCTX	None	41	19	60
	MID	30	17	47
	subMIC	72	49	121
ΔTEM	None	37	10	47
	MID	22	11	33
	subMIC	3	7	10

When ΔOXA was compared to the E. coli WT, not exposed to antibiotic and exposed to the MID of antibiotic, the number of proteins for which expression was significantly different increased from 18 to 48, respectively. On the other hand, ΔOXA maintained approximately the same number of proteins under the MID and subMIC conditions, which agrees with the variation in the number of differentially expressed proteins observed in comparison 1. ΔCTX exhibited higher numbers of proteins with differential expression than the E. coli WT not exposed to antibiotics, and even though a decrease in the number of differentially expressed proteins was observed upon exposure to the MID of antibiotic, exposure to subMIC conditions showed the greatest difference, with more than 100 proteins showing significantly higher or lower expression, with respect to the E. coli WT under subMIC conditions. Finally, ΔTEM showed a decreasing number of proteins, from conditions with no antibiotic to MID and subMICs, seemingly tending to be very similar to the profile of the E. coli WT with respect to protein expression levels when exposed to the highest concentration of antibiotic. The list of proteins detected at the subMIC of antibiotic in each knockout clone variant, as well as the fold changes data and statistics, is presented in Appendix S1, showing the pattern in expression of each protein under all test conditions (i.e., no antibiotic, MID, and subMIC).

Considering the number of proteins determined in both comparisons, ΔCTX showed the highest number of differentially expressed proteins, suggesting that the loss of this ESBL gene, among the three targeted resistance genes, caused the greatest stress to the bacterium and, thus, demanded the most intense adjustment of overall protein expression to overcome the impact of the antibiotic, at least, at the subMIC. The loss of *bla*_TEM-1_ was observed to have the least effect, as determined from the response of ΔTEM at the protein expression level, suggesting that *bla*_TEM-1_ is a less relevant gene for cephalosporin resistance, i.e., under the specific conditions used in this study.

### Impact of gene loss on the metabolism of ΔCTX, ΔOXA, and ΔTEM during exposure to antibiotics.

**FIG 3:**
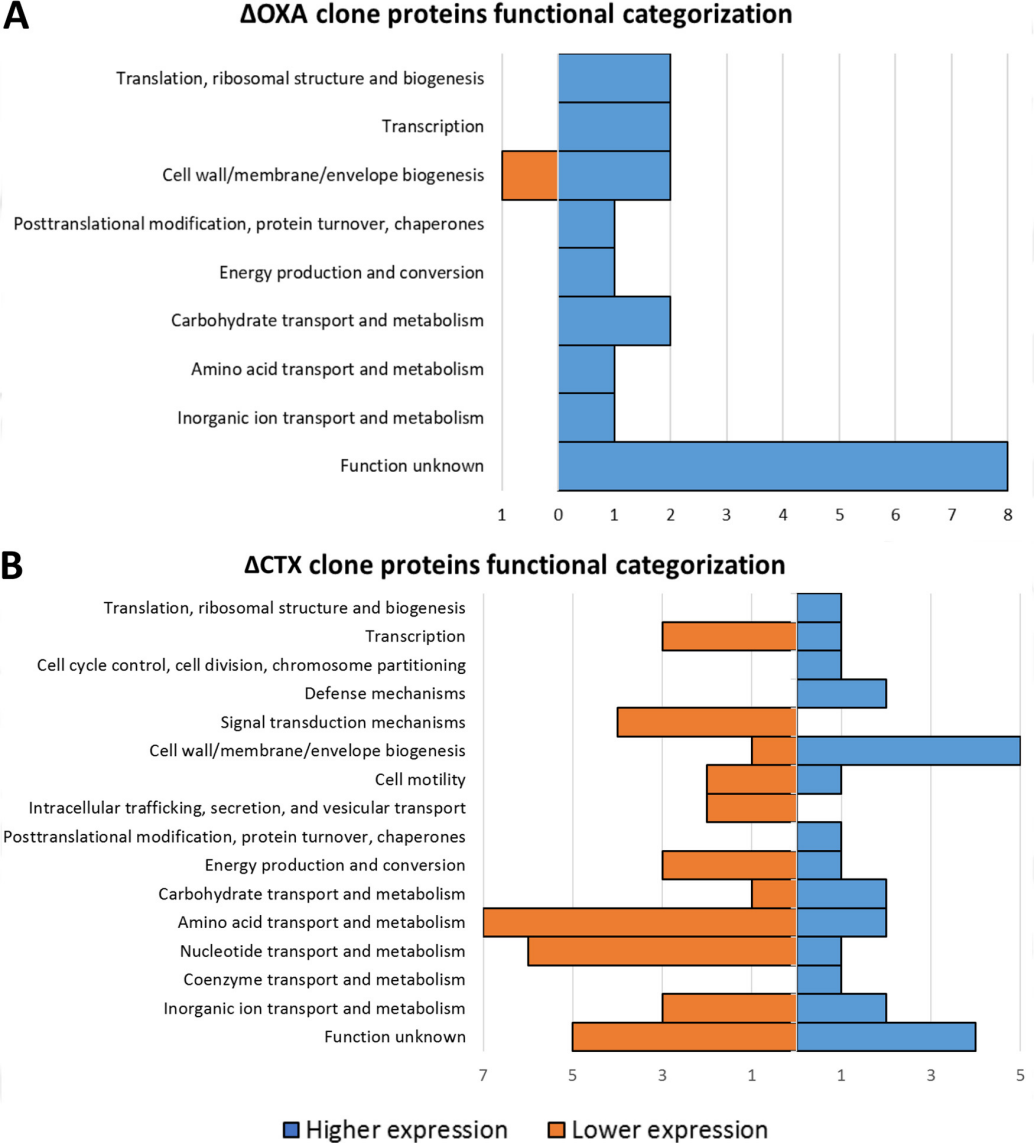
Functional categorization by COGs (Clusters of Orthologous Groups) of the different proteins with significantly higher or lower expression. (A) ΔOXA clone variant and (B) ΔCTX clone variant under subMIC conditions versus the same knockout clone variant with no antibiotic in each case. The *y* axis shows the functional category, and the *x* axis indicates the number of proteins displaying higher or lower expression for a certain COG category.

**FIG 4:**
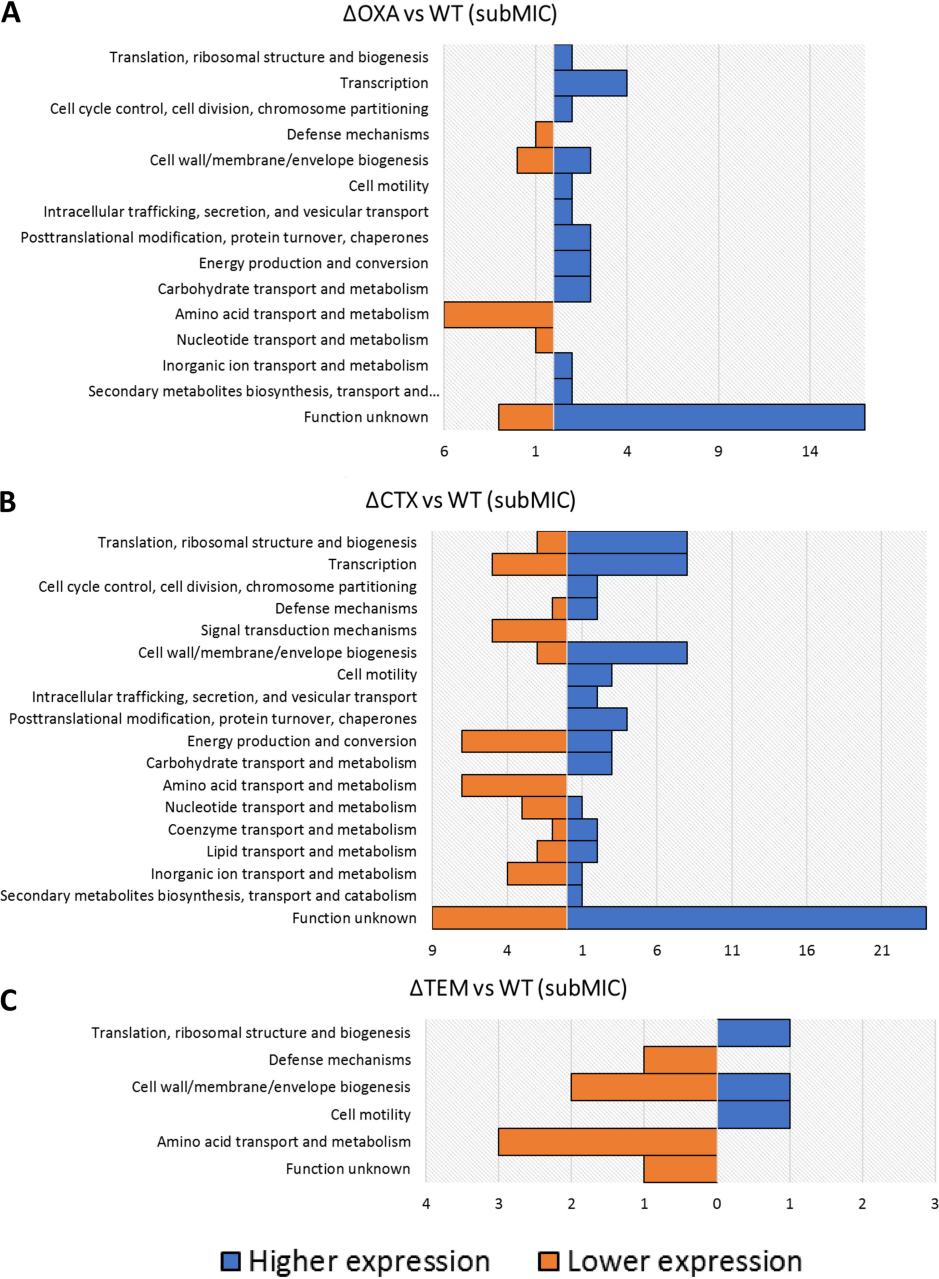
Functional categorization by COGs of the proteins with higher or lower expression for each of the clone variant in comparison 2. Clones ΔOXA, ΔCTX, and ΔTEM under subMIC conditions were compared with the E. coli WT at the same concentration. The *y* axis indicates the functional category; the *x* axis indicates the number of proteins displaying higher or lower expression for a particular COG category.

The proteins passing the set cutoffs under subMIC conditions in both clones and comparisons were categorized based on function (Fig. 3 and 4), in order to determine which aspects of the overall bacterial metabolism were being affected.

In comparison 1, the ΔCTX knockout clone variant showed “Function unknown” and “Cell wall/membrane/envelope biogenesis” to be the most represented categories, with “Function unknown” representing the highest number of proteins demonstrating significant differential expression (Fig. 3A). Regarding ΔOXA, functional categorization of these proteins showed that “Amino acid transport and metabolism,” “Nucleotide transport and metabolism,” and “Cell wall/membrane/envelope biogenesis” were the cellular traits most affected in general bacterial metabolism (Fig. 3B). In the case of “Amino acid transport and metabolism” and “Nucleotide transport and metabolism,” most proteins responded with lower expression, while in “Cell wall/membrane/envelope biogenesis,” related to the modification of the cell envelope, most proteins responded with higher expression. It is worth highlighting that “Function unknown” was the most identified functional category, together with “Amino acid transport and metabolism.”

When the same analysis was carried out using the results of comparison 2 with the ΔOXA clone variant, “Function unknown,” “Amino acid transport and metabolisms,” “Cell wall/membrane/envelope biogenesis,” and “Transcription” were observed to be the functional categories that showed the greatest number of changes in protein expression (Fig. 4A). The ΔCTX clone variant demonstrated changes in protein expression for “Function unknown,” “Cell wall/membrane/envelope biogenesis,” “Translation, ribosomal structure and biogenesis,” “Transcription,” and “Energy production and conversion,” as the most affected categories (Fig. 4B). It is worth highlighting that “Function unknown” was the category with the greatest number of proteins in both ΔOXA and ΔCTX. Generally, the number of differentially expressed proteins of ΔCTX (categorized into different functional categories) was higher than that of ΔOXA, suggesting that ΔCTX may compensate for the loss of this key resistance gene through a more intense response. ΔTEM was the knockout clone variant with the fewest changes in expressed proteins and the categories represented (Fig. 4C).

Overall, the functional categorization of the differentially expressed proteins detected represented functional features associated with transport and metabolism, cell wall/membrane/envelope biogenesis, transcription, or translation (among others). Similarly, previous studies have shown that proteins related to cell wall organization, transport, protein biosynthesis, or stress response, demonstrate differential expression after exposure to antibiotics (13). Metabolic adaptation allows the bacterial cells to optimize their metabolism and enhance protein synthesis to fulfill the metabolic needs to survive in the presence of the antibiotic (23). In this sense, functional categories, such as amino acid/nucleotide transport and metabolism, transcription, translation, transcription, or energy production, would be key factors to achieve the necessary metabolic adaptation. Other studies suggested that these adaptations are not exclusive to exposure to β-lactam antibiotics, as similar changes in metabolism or cell wall biosynthesis occur when bacteria are exposed to other kinds of antibiotics, such as tetracycline (24). The latter study suggests that E. coli shifts to a different set of cell wall biosynthesis proteins under antibiotic stress, as well as inducing several metabolic adaptations. Even though no specific common proteins among the present and cited studies have been identified, they agree with regard to the observations that metabolic adaptation, together with modifications in the cell wall and stress response, seems to be crucial in the response to antibiotic stress. Differences could be due to different species or antibiotics used in the different studies mentioned. Additionally, in both comparisons, i.e., each clone variant alone (comparison 1) and each clone variant compared with the E. coli WT (comparison 2) (Fig. 2), one of the major functional categories highlighted in all cases is proteins of unknown function, with several representatives differentially expressed in ΔOXA and ΔCTX. Further studies on the roles of these proteins are warranted.

### Differentially expressed proteins related to membrane, stress, gene expression, and antibiotic resistance.

When the results for ΔOXA and ΔCTX under the impact of the highest concentration of antibiotic (i.e., the condition under which the bacteria are stressed to the highest degree) were compared, several proteins related to the membrane, stress, and antibiotic resistance as well as proteins with unknown function were observed. Our results are in agreement with previous studies showing that ESBL-carrying bacteria express proteins involved in several aspects of cell metabolism, transport, and cell wall modification as well as stress proteins during antibiotic exposure (13, 14). Some proteins showed significant differences in expression in both clones (Table 6), although others were highlighted in only one of the clone variants, which could be related to the loss of the specific β-lactamase gene in each case. Generally, higher numbers of proteins in each category were found in comparison 2 (i.e., when each clone variant was compared with the E. coli WT at the same concentration of antibiotic, studying the knockout effects) than in comparison 1 (each clone variant at various concentrations of antibiotic) (Table 6).

**TABLE 6:** Proteins highlighted at subMIC of antibiotic compared with the same clone variant not exposed to antibiotics (comparison 1) and compared with the E. coli WT at the same concentration (comparison 2)

Protein group	Accession no.^*a*^	Description	FC for comparison			
			ΔCTX subMIC vs ΔCTX no antibiotic^*b*^	ΔCTX subMIC vs WT subMIC^*c*^	ΔOXA subMIC vs ΔOXA no antibiotic^*b*^	ΔOXA subMIC vs WT subMIC^*c*^
Hypothetical/DUF	QGA36222	Hypothetical protein FMA85_26960 (plasmid)	−1.69	−1.59		
	QGA34699	Hypothetical protein FMA85_19520		−1.73		
	QGA32310	DUF465 domain-containing protein		−1.58		
	QGA35305	DUF4092 domain-containing protein	−1.60	−1.66		
	QGA32870	DUF3102 domain-containing protein	−1.73	−1.74		
	QGA34857	DUF2756 family protein		3.10	1.68	1.87
	QGA31512	DUF2303 family protein	5.37	5.34		
	QGA32222	DUF1283 family protein		5.97		4.26
	QGA35173	DUF1090 domain-containing protein		4.27		3.20
	QGA35854	DUF2501 domain-containing protein	−1.66			
	QGA35694	DUF883 domain-containing protein	1.56			
	QGA32839	DUF882 domain-containing protein	−1.70			
	QGA31985	DUF2511 domain-containing protein				2.19
	QGA32890	DUF2190 family protein			1.86	2.42
Membrane/cell wall	QGA34355	PTS fructose-like transporter subunit IIB		1.84	1.75	1.90
	QGA33762	Peptidoglycan glycosyltransferase FtsI		1.59		
	QGA35582	MFS transporter	−1.73	−1.55		
	QGA32820	Membrane integrity-associated transporter subunit PqiC		−1.59		
	QGA33852	MDR efflux pump AcrAB transcriptional activator RobA		−1.73		
	QGA32136	Major outer membrane lipoprotein		7.28	3.33	4.30
	QGA31542	Major capsid protein		1.50		
	QGA31363	Long-chain fatty acid transporter FadL		−3.27	−2.26	−2.64
	QGA35082	Lipopolysaccharide ABC transporter substrate-binding protein LptA		1.84		1.72
	QGA32840	l,d-Transpeptidase	1.76	1.68		
	QGA32008	CNNM family cation transport protein YoaE	−1.64	−1.89		
	QGA33041	Arginine ABC transporter substrate-binding protein		−1.50		
	QGA33044	Arginine ABC transporter substrate-binding protein		−1.51		
	QGA31399	Histidine ABC transporter ATP-binding protein HisP	−1.6			
	QGA31396	Histidine ABC transporter substrate-binding protein HisJ	−1.54			
	QGA32815	Arginine ABC transporter substrate-binding protein				−2.09
	QGA33114	Bax inhibitor-1/YccA family protein	1.71	1.87		
Antibiotic	QGA34143	Cochaperone GroES		1.69	1.7	1.70
	QGA36195	Oxacillin-hydrolyzing class D beta-lactamase OXA-1 (plasmid)	2.28	2.69		−8.61
	QGA36164	Class A ESBL CTX-M-15 (plasmid)		−8.04		
	QGA36007	Class A broad-spectrum beta-lactamase TEM-1 (plasmid)	1.62	1.52		
	QGA35245	Antibiotic biosynthesis monooxygenase		1.93		1.73
Stress	QGA35625	RNA polymerase sigma factor RpoS		1.60		
	QGA34451	Fatty acid oxidation complex subunit alpha FadB		−1.59		
	QGA31976	DNA damage-inducible protein YebG		1.80		
	QGA33259	Transcription antiterminator/RNA stability regulator CspE		3.16		1.89
	QGA32001	Transcription antiterminator/RNA stability regulator CspE		2.98	1.76	1.81
	QGA34530	Thioredoxin TrxA		2.37		
	QGA34154	Lysine decarboxylase CadA		−1.94		−1.74
	QGA35118	DEAD/DEAH family ATP-dependent RNA helicase	2.24	1.90		
	QGA34268	CsbD family protein		3.47		3.52
	QGA31167	Autonomous glycyl radical cofactor GrcA		1.58	1.55	
	QGA32723	Anti-sigma-28 factor FlgM		3.72		3.47
	QGA34791	Acid-activated periplasmic chaperone HdeB		2.58	2.28	1.91
	QGA31717	YoeB-YefM toxin-antitoxin system antitoxin YefM		2.31		2.15
	QGA35950	Type II toxin-antitoxin system Phd/YefM family antitoxin (plasmid)		3.42		2.88
	QGA32789	Addiction module toxin, GnsA/GnsB family		7.81		7.81
Others	QGA35923	YgdI/YgdR family lipoprotein	1.50	1.67		
	QGA32482	YciI family protein		2.38		2.62
	QGA35487	Putative selenate reductase subunit YgfK	−1.61	−1.54		
	QGA31547	Phage tail protein	2.03	1.88		
	QGA32888	Peptidase	−1.80	−1.87		
	QGA36198	Mobilization protein (plasmid)		−1.56		
	QGA36220	MobC family plasmid mobilization relaxosome protein (plasmid)	−1.70	−1.50		1.60
	QGA34451	Fatty acid oxidation complex subunit alpha FadB		−1.59		
	QGA34297	Dipeptidase PepE		−2.19		
	QGA34143	Cochaperone GroES		1.69	1.7	1.70
	QGA33763	Cell division protein FtsL	1.72	1.86		
	QGA32410	Bifunctional anthranilate synthase glutamate amidotransferase component TrpG/anthranilate phosphoribosyltransferase TrpD	−1.64	−2.35		−4.61
	QGA33114	Bax inhibitor-1/YccA family protein	1.71	1.87		
	QGA35038	Acetyl coenzyme A carboxylase biotin carboxyl carrier protein		1.57		
	QGA36206	MobC family plasmid mobilization relaxosome protein (plasmid)				−2.66
	QGA36199	MbeD family mobilization/exclusion protein (plasmid)				−3.35
	QGA34370.1	50S ribosomal protein L31		2.03		
	QGA32706.1	50S ribosomal protein L32			1.63	

a NCBI protein accession numbers.

b Comparison 1.

c Comparison 2.

The membrane surrounding the bacterial cell is the structure in direct contact with the environment, and changes in the membrane structure and composition, including changes in the expression of key membrane proteins, are critical for the bacterium and its interaction with the environment (25). These changes can be related to membrane permeability, for example, to restrict entry of an antibiotic into the cell, or more specialized changes focused on the expulsion of an antimicrobial agent (26, 27). A total of 16 differentially expressed membrane-related proteins in ΔCTX were observed, while only 5 were observed in ΔOXA. Overall, they represent transporters, porins, a multidrug-efflux pump, cell envelope and peptidoglycan modification proteins, and membrane integrity proteins. Four proteins in this group exhibited differential expression in both knockout clone variants. In the case of ΔOXA, three of these shared proteins were detected in both comparisons, although in ΔCTX, they were found only in the comparison with the E. coli WT (Table 6).

Generally, the fold changes in expression of these proteins detected at the same time in both ΔCTX and ΔOXA showed more pronounced differences in ΔCTX than in ΔOXA. Overall, this suggests that the number of proteins and the fold changes in expression may need to be higher in ΔCTX than in ΔOXA, i.e., in order to try to compensate for the respective β-lactamase gene loss. As an example, a major outer membrane lipoprotein (GenBank accession no. QGA32136.1) demonstrated an increased expression almost 8.0-fold greater in the ΔCTX mutant than in the E. coli WT, while in ΔOXA, expression of the same protein was only 4.3-fold greater than in the E. coli WT. Differential expression in outer membrane proteins and lipoproteins in response to the presence of antibiotics was reported previously (13, 28). These changes have been suggested to be important, since outer membrane proteins and peptidoglycan-associated proteins have essential roles in transport, cell wall integrity maintenance, or adhesion (29, 30). Mobilization of these proteins may be directed to the structural strengthening of the bacterial envelope to face the presence of β-lactam antibiotics (13).

Additionally, several transport proteins were detected in both ΔCTX and ΔOXA. Transport-related proteins are crucial for survival of cells, since they control the traffic of a variety of metabolites through the membrane, adjusting the intake or expulsion capacity to the specific condition encountered. Previous studies have shown increased expression of several of these proteins (13), although all transport-related proteins highlighted in Table 6, except the phosphotransferase system (PTS) fructose-like transporter subunit IIB, demonstrated a lower expression. This may indicate a different type of adaptation in the specific experimental set used in the present work. One important protein is the l,d-transpeptidase, with expression higher in ΔCTX (fold change of 1.76 in comparison 1 and 1.68 in comparison 2). This enzyme is involved in the peptidoglycan biosynthesis, being responsible for generating the so-called 3→3 links. These cross-links are not the most common ones in the peptidoglycan (31). Instead, the typical links are the 3→4 links performed by the d,d-transpeptidase. Antibiotics of the β-lactam family (penam and cephem classes) are able to inhibit the d,d-transpeptidase activity (32), but they do not inhibit l,d-transpeptidase efficiently. Overproduction of l,d-transpeptidase in the presence of antibiotics would make it possible to bypass the step inhibited by the antibiotic and ensure the integrity of the peptidoglycan (33). This l,d-transpeptidase is overexpressed only in the ΔCTX clone variant, in which, theoretically, the stress is much higher due to the main defense against cephalosporins having been knocked out. Our analysis thus allows detection of alternative mechanisms of reduced susceptibility, which may go unnoticed when classical methods of resistance mechanism detection are used.

Regarding the ΔOXA clone variant, the level of expression of this specific protein is similar to that of the E. coli WT, as it seems not necessary to increase expression because of the presence of *bla*_CTX-M-15_, which effectively acts against the cephalosporin. Additionally, one membrane-associated protein (GenBank accession no. QGA32815) was detected and differentially expressed only in ΔOXA compared with the E. coli WT (Table 6).

Other membrane proteins, such as the efflux pump AcrAB and the relevant porins (OmpA, OmpC, OmpX, OmpT, and the regulator OmpR), were also checked. All of the mentioned proteins were detected in all clones, although the fold changes (FCs) did not indicate any marked differences in expression in any of the comparisons performed. In any case, the *P* values associated with these proteins in the present analysis were over the threshold of 0.05 and, hence, considered not statistically significant. OmpC and OmpF are two major porins present in E. coli that have been related to antibiotic resistance (34, 35). OmpF is considered one of the main pathways for antibiotic import, and reduced or no expression of this porin is commonly seen in antibiotic-resistant E. coli strains (34, 36, 37), which may be the case in the studied strain. The porin OmpC, though it does not show significant differences in its expression, does contain several mutations that introduce amino acid changes in comparison with strain E. coli ATCC 25922 (accession no. CP037449.1) (D47N, V50E, A85S, P86A, S88N, I106V, Q173K, S178D, K188G, V210I, W221D, N225F, T232E, G233R, L234Y, I235L, T237N, V311I, A312N, Q252R, and A356D; insertion of 8 amino acids at position 180 and deletion of 6 at positions 226 to 231). Mutations in this porin have been linked to antibiotic resistance (35, 38). It may be the case that these mentioned mutations in OmpC provide resistance against antibiotics and, hence, it is not necessary to modify its expression. However, this should be analyzed and demonstrated in future studies.

Regarding the antibiotic resistance-related genes, overexpression of *bla*_OXA-1_ and *bla*_TEM-1_ was observed in ΔCTX. The *bla*_OXA_ type β-lactamases are described as particularly active against oxacillin (an antibiotic used mainly for Gram-positive infections), although activities against other penicillins also have been detected, and in some cases, OXA type β-lactamases are able to attack cephalosporins and carbapenems (39–41). According to the β-lactamase database (42), *bla*_OXA-1_ shows high activity against oxacillin and ampicillin, as well as some degree of activity against cefaloridine, a first-generation cephalosporin (the same generation as cefadroxil). The *bla*_TEM-1_ gene product is considered a prevalent β-lactamase among Gram-negative bacteria which acts against penicillins but also has some degree of activity against narrow-spectrum cephalosporins (such as cephalothin or cefazolin) but not extended-spectrum cephalosporins (43, 44). Low activities of both enzymes against cephalosporins of the same generation as cefadroxil would explain their overexpression in ΔCTX, i.e., as an attempt to compensate for the loss of *bla*_CTX-M-15_, the gene that confers resistance against cephalosporins. Expression of both *bla*_CTX-M-15_ and *bla*_OXA-1_ or both *bla*_CTX-M-15_ and *bla*_TEM-1_ was detected in ΔTEM and ΔOXA, respectively. However, no significant changes in their expression were noticed, probably due to the presence of *bla*_CTX-M-15_ in both cases. Additionally, the chromosomal β-lactamase AmpC is also present in E. coli CCUG 70778 and, consequently, in the three mutants. This β-lactamase was expressed in all cases, although the FCs were not substantial in any of the comparisons, and the corresponding *P* values were also poor.

Considering the results of comparisons 1 and 2, a total of 15 stress-related proteins were differentially expressed (Table 6), of which all 15 were detected in ΔCTX, one of them in both comparisons and the rest only when compared with the E. coli WT. On the other hand, a total of 10 proteins were found in the ΔOXA clone, from which one of the proteins was seen in comparison 1 but not in the comparison with respect to the E. coli WT (comparison 2), and two of them were found in both comparisons. Three of the proteins shared by both clone variants belong to toxin-antitoxin systems (TAs), two chromosomally encoded (antitoxin YefM [QGA31717.1] and the addiction module toxin [GnsA/GnsB family] [QGA32789.1]) and one whose gene is located in pSUH-1, among other stress-related proteins. These systems are important in situations of nutritional or environmental stress, for example, acting as modulators of cell growth to overcome stressful situations (45–48). A higher expression of TAs in the presence of antibiotics has previously been reported, such as the chromosomal *mazEF* system (13). In this case, it is suggested that *mazEF* is part of the programmed cell death response (49, 50) that leads to the death of most of bacterial cells but allows the survival of a small fraction of the population. Nevertheless, this phenomenon is not yet fully understood. One of the most significant stress-related proteins differentially expressed in ΔCTX was the RNA polymerase sigma factor RpoS (QGA35625.1), which regulates the expression of hundreds of genes involved in stress management, among other functions (51). Another protein, YebG (QGA31976.1), is considered an uncharacterized protein for which the specific function is unknown, although it is sometimes described as a DNA damage-inducible gene of the SOS regulon (52). Additionally, some studies performed on E. coli indicated that the overexpression of *yebG* is involved in the increase in resistance to the biocide polyhexamethylene biguanide (52–54).

Some proteins are related to gene expression, such as the DEAD/DEAH family ATP-dependent RNA helicase (QGA35118.1), which is differentially expressed only in ΔCTX. This could be related to a higher demand for gene expression. Another protein is the anti-sigma-28 factor FlgM (QGA32723.1) (overexpressed in both clone variants), which inhibits the sigma factor 28, avoiding the expression of flagellar genes (55, 56). Downregulation of flagellar genes after exposure to antibiotics may lead to the emergence of a biofilm-like state, enabling the bacterium to survive in the presence of antibiotics (11). Ribosomal proteins have also been reported to exhibit an increase in expression upon exposure to antibiotics. A hypothesis is that they may be related to the necessity of an enhanced and efficient translation machinery to facilitate the necessary adjustments in the presence of the antibiotic stress (12, 13). In the present work, two overexpressed ribosomal proteins were highlighted under subMIC conditions: 50S ribosomal protein L31 (ΔCTX, comparison 2) and 50S ribosomal protein L32 (ΔOXA, comparison 1).

Finally, we detected hypothetical proteins and proteins with domain of unknown function (DUF) with significant variation in their expression in both analyses performed with ΔCTX and ΔOXA (Table 6). A higher number of these proteins were detected in ΔCTX than in ΔOXA, most of them detected when compared with the E. coli WT. The proteins of this group demonstrated significant differential expression in both clones, tending to exhibit greater changes in expression in ΔCTX (Table 6). These are proteins for which a function has not been described or is not yet clear. These results are important, since they confirm that these proteins are actually expressed by the cell and may have a role in regulation of AMR. Studies to further clarify the roles of these hypothetical and DUF proteins, as well as the proteins identified in this work, in a protein network for response to antibiotic pressure are needed in order to understand their roles in phenotypic resistance modulation.

### Conclusions.

Here, we provide a protocol for studying the impact of resistance gene loss on protein expression in clinical strains under antibiotic selection pressure. The highest number of differentially expressed proteins was observed in ΔCTX, suggesting that this knockout mutant may require more intense adjustments in the protein network to compensate for the loss of the resistance gene for dealing with exposure to antibiotics. The present study was able to detect alternative mechanisms of reduced susceptibility, such as overexpression of l,d-transpeptidase, which may go unnoticed using classical methods of resistance mechanism detection, and proves the effectiveness of the protocol of homologous recombination for the targeted gene knockout of selected plasmid-borne antibiotic resistance genes. Our study provides a framework for studying the effect of targeted loss of resistance genes on the global protein expression in multiresistant strains and identifies proteins that could be the bases of subsequent studies to define and prove their roles in the process. Further, in-depth analyses are needed to determine the possible specific role of each protein in antibiotic resistance.

## MATERIALS AND METHODS

### Strains and culture conditions.

Escherichia coli strain CCUG 73778 (E. coli WT) is a virulent, multiresistant ESBL-producing ST131-O25b-type strain, which was isolated during an outbreak at the Sahlgrenska University Hospital (Gothenburg, Sweden) in 2008 (57–59). The genome sequence of E. coli CCUG 73778 has been determined, resulting in a complete and closed sequence of a single chromosome and six plasmids; two large plasmids, pSUH-1 and pSUH-2, carry the β-lactamase genes *bla*_OXA-1_ and *bla*_TEM-1_ and the ESBL gene *bla*_CTX-M-15_, and the strain has other resistance factors that are both plasmid and chromosomally encoded (57). Escherichia coli MFDpyr is a donor strain used for conjugation which is deficient in the production of diaminopimelic acid (DAP) (60). The E. coli WT was cultivated on blood agar medium (Columbia agar base plus 5% defibrinated horse blood). E. coli MFDpyr was plated on LB agar medium supplemented with 300 μM DAP. Both strains were incubated at 37°C overnight under aerobic conditions.

### Generation of knockout clone variants of the E. coli WT.

Knockouts, or targeted elimination, of specific genes (*bla*_CTX-M-15_, *bla*_OXA-1_, and *bla*_TEM-1_) were performed using the meganuclease I-SceI deletion system described previously (61, 62). The plasmids used were the nonreplicative plasmid pSEVA-328s and the meganuclease I-SceI-encoding pSEVA-528s (61, 63), which encode chloramphenicol (Cm) and tetracycline (Tet) resistance gene markers, respectively.

**(i) Primer design.** Primer pairs for the amplification of 500 bp upstream (homologous region S1) and downstream (homologous region S2) of the targeted genes were designed, following the recommendations of a previously described protocol (61). Restriction sequences were introduced in the 5 ends of primers S1-F (EcoRI) and S2-R (BamHI or HindIII). Primers were tested with fastPCR (64, 65) and the NEBbuilder online tool (http://nebuilder.neb.com) to ensure specificity and compatibility between pairs. Primer sequences are listed in Table S2.

**(ii) Generation of the S1-S2 fragment.** The regions S1 and S2 of the targeted genes were amplified, using Q5 hot-start high-fidelity 2× master mix, following the recommendations of the manufacturer (New England Biolabs, Ipswich, MA, USA). The annealing temperature was set to 60°C and elongation time to 1 min. PCR products were purified by agarose gel electrophoresis and gel band extraction, using Illustra GFX PCR DNA and gel band purification (GE Healthcare, Chicago, IL, USA). The resulting samples were quantified, using NanoDrop 2000c spectrophotometer (Thermo Fisher Scientific, Waltham, MA, USA). The S1 and S2 amplicons for each gene were joined by overlap extension PCR (OE-PCR) (66), a two-step PCR, wherein the first step joins the two target segments (S1 and S2) and the second step amplifies the entire sequence (S1-S2 segment). Using the Q5 hot-start high-fidelity 2× master mix, the first-step PCR was performed with a total volume of 25 μL, adding 100 ng of the larger fragment and an equimolar quantity (1:1 ratio of S1 and amplicon S2) of the smaller fragment, with an annealing temperature of 60°C and an elongation time of 1 min. No primers were added, and the PCR was performed for 15 cycles. After this, 25 μL of the PCR mixture from the Q5 hot-start high-fidelity 2× master mix, including the primers S1-forward and S2-reverse, but without DNA, was prepared and added to previous PCR mixture, making the final volume 50 μL. The annealing temperature was set at 60°C, the elongation time was 1 min, and 35 cycles were performed. The PCR products of expected size were purified by gel band extraction as described above.

**(iii) Construction of the three pSEVA-328s variants.** Extraction of plasmids pSEVA-328s (host, E. coli λ*pyr* strain) and pSEVA-528S (host, E. coli DH5α) were performed, using the 1× Zyppy plasmid miniprep kit (Zymo Research, Irvine, CA, USA). Plasmid pSEVA-328S and the products of the OE-PCR were digested with EcoRI-HF and BamHI-HF in the cases of the *bla*_OXA-1_ and *bla*_CTX-M-15_ S1-S2 fragments and EcoRI-HF and HindIII in the case of the *bla*_TEM-1_ S1-S2 fragment, following the instructions of the manufacturer (New England Biolabs, Ipswich, MA, USA). Digestion products were purified by gel band extraction as described above. Quantities equivalent to a vector/insert ratio of 1:3 were used for ligation, using the T4 DNA ligase (Invitrogen, Carlsbad, CA, USA), following the recommendations of the manufacturer.

**(iv) Transformation into E. coli MFDpyr.** Chemically competent cells of E. coli MFDpyr were prepared using a variation of the method of Cohen et al. (67). E. coli MFDpyr was inoculated into a 5-mL LB tube supplemented with 0.3 mM DAP and incubated overnight at 37°C, with shaking (200 rpm). A total of 500 μL of the overnight culture was transferred into a 250-mL Erlenmeyer flask containing 100 mL of sterile LB supplemented with 0.3 mM DAP. The culture was incubated at 37°C, with shaking (200 rpm), until the optical density (OD) reached 0.5 (2 to 4 h). The cells were harvested, collected in a 50-mL Corning tube, and incubated on ice for 30 min. Cells were harvested by three successive centrifugations at 3,000 × *g* for 15 min at 4°C, carefully removing the supernatant after each centrifugation. Cells were resuspended with 50 and 5 mL of ice-cold 0.1 M CaCl_2_ for the second and third centrifugations, respectively. Finally, cells were resuspended in 1.6 mL of ice-cold 0.1 M CaCl_2_ and 0.4 mL of 40% glycerol. Aliquots of 50 μL were stored immediately at −80°C. Aliquots of 50 μL of chemically competent cells were thawed on ice and used for transformation, following the heat shock protocol described by New England Biolabs (https://international.neb.com/protocols/2012/05/21/transformation-protocol). In this case, 950 μL of LB broth supplemented with 0.3 mM DAP was used. Recovered clones were confirmed by colony PCR, using the pMG-1 and pMG-2 primers (Table S2), which flank the insert section of the plasmid. PCR products were confirmed by Sanger sequencing (Eurofins Scientific, Luxemburg). Confirmed clones were stored at −80°C in 20% glycerol until further analysis.

**(v) Deletion of genes and confirmation of the knockout clone variants.** One colony of the donor strain, E. coli MFDpyr (carrying pSEVA-328 with the S1-S2 fragment), was inoculated onto new LB agar medium with 0.3 mM DAP, in a circle with a 1-cm diameter. One colony of the recipient strain, the E. coli WT, was spread over the donor strain. The culture was incubated for 18 h at 37°C. All biomass was collected from the culture plates and resuspended in 1 mL of phosphate-buffered saline (PBS). Serial dilutions were prepared, and 100 μL of 10^−4^, 10^−5^, and 10^−6^ dilutions were plated, in duplicate, on LB agar medium supplemented with Cm (30 μg/mL) for selection of the clones with inserted plasmid. A total of 5 to 10 clones were recovered and used for the second conjugation, in which the process was repeated, using the donor strain, E. coli MFDpyr, with the plasmid pSEVA-528s. After resuspension of the cells in PBS and serial dilutions, 10^−4^, 10^−5^, and 10^−6^ dilutions were plated, in duplicate, on LB agar medium supplemented with Tet (10 μg/mL). Cultures were incubated overnight at 37°C under aerobic conditions.

**FIG 5:**
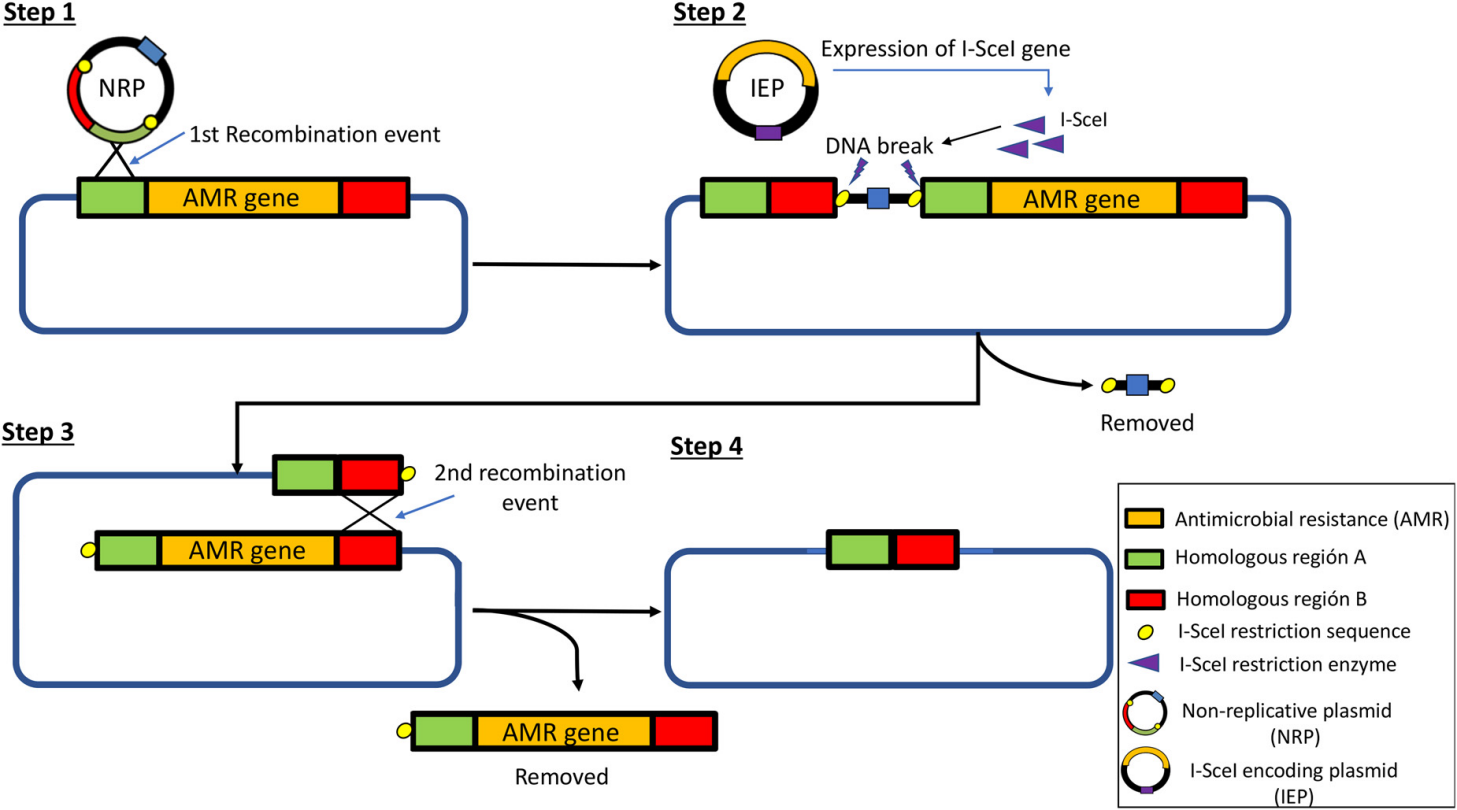
Different steps of the two plasmids homologous recombination system for knockout of targeted genes, described by Aparicio et al. (61). Step 1 shows the insertion of the nonreplicative plasmid (NRP) in the targeted DNA sequence through the first homologous recombination event, step 2 shows the action of the I-SceI-encoding plasmid over the NRP inserted next to the targeted gene, step 3 shows the second recombination event that results in the removal of the targeted gene from the genome, and step 4 shows the result after DNA repair of the targeted sequence.

Several clones were recovered in new LB agar medium with Tet (10 μg/mL) and then inoculated into 5 mL liquid LB supplemented with Tet (10 μg/mL) and 1 mM 3 methyl-benzoate (3-MBz) to induce the expression of the meganuclease I-SceI. The tube was incubated overnight at 37°C with shaking (200 rpm). A volume of 100 μL of the overnight culture was used to prepare serial dilutions in PBS. A total of 100 μL of 10^−4^, 10^−5^, and 10^−6^ dilutions was plated, in duplicate, on LB agar medium, which was incubated at 37°C overnight. An overview of the molecular process is presented in Fig. 5. Clones that were able to grow on LB but did not grow on Cm were selected for the next step. Elimination of pSEVA-528s from the selected clones was performed, cultivating as many as 2 or 3 times on LB agar medium without Tet. Knockout clone variants were confirmed by colony PCR, using primers designed to target outside the gene sequence, the OUT-F and OUT-R primers (Table S2), and Sanger sequencing. Additional confirmations of the removal of the targeted genes were performed by PCR, using internal primers previously described for each gene (68).

### DNA extraction and sequencing.

Total DNA extractions were performed with 1 transfer loop of fresh biomass of confirmed clones, using the Wizard genomic DNA purification kit (Promega, Madison, WI, USA). Quality and quantity of DNA were assessed by NanoDrop (quality ratios) and the Qubit double-stranded-DNA (dsDNA) broad range (BR) assay kit (Invitrogen, Carlsbad, CA, USA). DNA samples of each knockout clone variant were submitted for whole-genome Illumina sequencing (Eurofins Scientific, Luxemburg). Briefly, the DNA libraries were prepared using an optimized protocol and standard Illumina adapter sequences. Sequences were determined using Illumina NovaSeq (read mode, 2 × 150 bp). Illumina sequence reads were mapped against the reference genome of E. coli CCUG 73778 (57), using the CLC-Genomics Workbench (Qiagen, Hilden, Germany), for confirmation of the complete removal of the targeted genes in each case.

### Phenotypic antibiotic resistance analysis.

MIC determinations for 27 antibiotics (listed in Table 1) were performed at the National Reference Laboratory for Antibiotic Resistance (Växjö, Sweden). which follows the guidelines of the European Committee on Antimicrobial Susceptibility Testing (EUCAST), according to the ISO standard 20776-1:2019 (69). Briefly, broth microdilutions were done with Mueller-Hinton broth, inoculating 5 × 10^5^ CFU/mL. Incubations were in sealed panels at 35 ± 1°C for 18 ± 2 h under aerobic conditions. The recommended antibiotic panel for *Enterobacteriaceae* was selected and determinations of resistance or sensitivity were performed following the EUCAST MIC breakpoint tables for determination of resistance and sensitivity, v12.0 2022 (www.eucast.org/clinicalbreakpoints). Cefadroxil (CFR) was tested in house, following the same protocol.

Disk diffusion tests were performed following the EUCAST standardized disk diffusion method (https://www.eucast.org/ast_of_bacteria/disk_diffusion_methodology/). Briefly, cell suspensions of a McFarland 0.5 standard were prepared in PBS and spread onto Mueller-Hinton agar plates. Antibiotic discs were placed onto the plates and incubated at 35 ± 1°C for 18 ± 2 h under aerobic conditions. Determinations of resistance or sensitivity were done, following the EUCAST zone diameter breakpoint tables (v12.0, 2022).

### Preparation of samples for proteomic analysis.

The knockout clone variants (E. coli ΔOXA, E. coli ΔCTX, and E. coli ΔTEM) and the E. coli WT were cultured on Columbia agar medium supplemented with 5% of defibrinated horse blood under aerobic conditions at 37°C for 24 h. Fresh biomass (1 or 2 colonies) was collected to prepare a 0.5 McFarland cell suspension in PBS, using a McFarland densitometer (DEN-1 BioSan; ProfilLab24 GmbH, Berlin, Germany). A volume of 100 μL from the 0.5 McFarland suspension was diluted in PBS to a final volume of 2,000 μL (dilution 1:20), obtaining the bacterial working solution (BWS). Afterward, 400 μL of the BWS was transferred, in triplicate, to 4 mL Mueller-Hinton broth (MHB), according to the antibiotic conditions to be tested, i.e., subMIC, MID (half of the subMIC), and no antibiotic, in triplicate, in each case. Cefadroxil (stock,5 mg/mL) was used at concentrations of 8 μg/mL (highest concentration of antibiotic, i.e., subMIC), 4 μg/mL (middle concentration of antibiotic, i.e., MID), and no added antibiotic. Samples with and without antibiotic were mixed and incubated in aerobic conditions for 18 h at 37°C with shaking (200 rpm). Thereafter, 1 mL of sample was taken from each tube and centrifuged at 10,000 × *g* for 5 min. Precipitated cells were washed with 1 mL PBS and centrifuged at 10,000 × *g* for 5 min. Following three washing steps with PBS under the same conditions, the final pellet was resuspended in 1 mL PBS and OD was adjusted to 1.0. Samples were centrifuged under the same conditions and the final pellet was resuspended in 150 μL of PBS before being transferred to 0.2-μL tubes with acid-washed glass beads (150 to 212 μm; Sigma-Aldrich, Inc., Darmstadt, Germany). Then, 15 μL of 20% SDS (sodium dodecyl sulfate solution) was added, and samples were mixed by inverting the tubes. Samples were treated by bead beating for 5 min at 1/25 frequency by means of a bead beater (TissueLyser II; Qiagen, Hilden, Germany) and centrifuged again at 100 × *g* for 1 min. Supernatant was transferred to new tubes (1.7-mL Axygen maximum recovery tubes) with 100 μL of 2% SDS. Samples were mixed by inverting, and the supernatant, without glass beads, was transferred carefully to a new tube and stored at −20°C until further analysis. Protein concentrations were determined, using a Pierce bicinchoninic acid (BCA) protein assay kit (Thermo Fisher Scientific, Waltham, MA, USA) and the Benchmark Plus microplate reader (Bio-Rad Laboratories, Hercules, CA, USA) with bovine serum albumin (BSA) solutions as standards.

### Protein digestion and TMT labeling.

Aliquots containing 30 μg of protein from each sample and references (prepared by mixing equal protein amounts of each sample) were digested with trypsin, using the filter-aided sample preparation (FASP) method (70). Briefly, samples were reduced with 100 mM dithiothreitol at 60°C for 30 min, transferred to 30-kDa-MWCO (molecular-weight-cutoff) Nanosep 30k Omega filters (Pall Corporation, Port Washington, NY, USA), washed repeatedly with 8 M urea and once with digestion buffer 1% sodium deoxycholate [SDC] in 50 mM triethylammonium bicarbonate [TEAB] prior to alkylation, with 10 mM methyl methanethiosulfonate in digestion buffer, for 30 min. Digestion was performed in digestion buffer by addition of 0.3 μg Pierce MS-grade trypsin (Thermo Fisher Scientific) at 37°C and incubation overnight. An additional portion 0.3 μg of trypsin was added, and the mixture was incubated for another 2 h. Peptides were collected by centrifugation at 10,000 × *g*.

Digested peptides were labeled, using TMT 10-plex isobaric mass tagging reagents (Thermo Scientific), according to the instructions of the manufacturer. Samples and references were combined into four TMT sets. Sodium deoxycholate was removed by acidification with 10% trifluoroacetic acid (TFA). The combined sets were separated into 40 fractions with basic reversed-phase chromatography (bRP-LC), using a Dionex Ultimate 3000 ultra-high-performance LC (UPLC) system (Thermo Fisher Scientific). Peptide separations were performed using a reversed-phase XBridge BEH C_18_ column (3.5 μm, 3.0 by 150 mm; Waters Corporation), and a linear gradient was generated, mixing solvent A (10 mM ammonium formate buffer at pH 10.00) and solvent B (90% acetonitrile, 10% 10 mM ammonium formate at pH 10.00), increasing solvent B from 3% to 40% over 18 min, increasing to 100% solvent B over 5 min, and finally staying at 100% solvent B for 5 min. All steps were performed at a flow rate of 0.4 mL/min. The 40 fractions were concatenated into 10 fractions (1 + 11 + 21 + 31, 2 + 12 + 22 + 32, …10 + 20 + 30 + 40), dried, and reconstituted in 3% acetonitrile, 0.2% formic acid.

### LC-MS/MS.

The fractions were analyzed using an Orbitrap Fusion Lumos Tribrid mass spectrometer (MS) interfaced with an Easy-nLC1200 liquid chromatography system (Thermo Fisher Scientific). Peptides were trapped on an Acclaim Pepmap 100 C_18_ trap column (100 μm by 2 cm; particle size, 5 μm; Thermo Fisher Scientific) and separated on an in-house packed analytical column (75 μm by 35 cm; particle size, 3 μm; Reprosil-Pur C_18_ [Dr. Maisch]), using a linear gradient from 5% to 33% solvent B over 77 min followed by an increase to 100% solvent B for 3 min and then 100% solvent B for 10 min at a flow rate of 300 nL/min. Solvent A was 0.2% formic acid, and solvent B was 80% acetonitrile, 0.2% formic acid. Precursor ion mass spectra were acquired at 120,000× resolution, and MS/MS analysis was performed in a data-dependent multinotch mode, wherein collision-induced dissociation (CID) spectra of the most intense precursor ions were recorded in ion trap at a collision energy setting of 35 for 3 s (top speed setting). Precursors were isolated in the quadrupole with a 0.7 *m*/*z* isolation window, charge states 2 to 7 were selected for fragmentation, and dynamic exclusion was set to 45 s and 10 ppm. MS3 spectra for reporter ion quantitation were recorded at 50,000× resolution with high-energy collisional dissociation (HCD) fragmentation at a collision energy of 65, using the synchronous precursor selection.

### Proteomic data analysis.

The data files for each set were merged for identification and relative quantification, using Proteome Discoverer version 2.4 (Thermo Fisher Scientific). The search was made against all protein sequences encoded by the genome sequence of Escherichia coli CCUG 73778 (57), using Mascot version 2.5.1 (Matrix Science, London, United Kingdom) as a search engine. The precursor mass tolerance was set to 5 ppm and fragment mass tolerance to 0.6 Da. Tryptic peptides were accepted with zero missed cleavage, variable modifications of methionine oxidation, and fixed cysteine alkylation; TMT label modifications of N-terminal and lysine were selected. The reference samples were used as denominators and for calculation of the ratios. Percolator (71) was used for the validation of identified proteins. TMT reporter ions were identified in the MS3 HCD spectra with 3 milli-mass units (mmu) mass tolerance, and the TMT reporter intensity values for each sample were normalized to the total peptide amount. The quantified proteins were grouped by sharing the same sequences to minimize redundancy. Only the values for the unique peptides were used for quantification.

### Proteomic data processing.

Principal-component analysis was performed using R v4.0.3. Briefly, protein expression values were log_2_ transformed. The prcomp function from the stats package was used to run a principal-component analysis, which was then plotted using ggplot2. Two different comparisons of protein expression were done. In the first comparison (Fig. 2, comparison 1), fold changes were calculated for each strain individually by dividing the mean abundance of every protein from growth at MID or subMIC antibiotic concentrations by the mean abundance of the same protein from growth with no antibiotic. *P* values were calculated from the log_2_-transformed value of protein abundances at MID or subMIC antibiotic concentrations with respect to the knockout clone variant without antibiotic. For this purpose, Welch’s *t* test was applied, using the TTEST function in Excel, with a two-tail distribution and two-sample unequal variance. Volcano plots were generated in Excel by plotting the fold change in expression (log_2_) against the corresponding significance *P* value (−log_10_). Final values were filtered, using the thresholds of a *P* value of ≤0.05 and fold changes of ≥1.5 (proteins with significantly higher expression) and ≤−1.5 (proteins with significantly lower expression). In the second comparison (Fig. 2, comparison 2), the same calculations were performed but comparing the expression in each knockout clone variant with that in the original E. coli WT strain at fixed concentrations of antibiotics. In both cases, only proteins for which more than 1 peptide was detected were considered for further analysis. The final lists of proteins after filtering were analyzed, using eggNOG-mapper v2 (72) for functional categorization.

### Data availability.

The data sets produced in this study are available in the following databases. Proteomic MS data can be found at the ProteomeXchange with identifier PXD029140. Raw Illumina reads of the clone variants can be found in the GenBank Sequence Read Archive (SRA): SRR15929430 (ΔOXA), SRR15929429 (ΔCTX), and SRR15929428 (ΔTEM).
