# Supplementary material for: Knockout of Targeted Plasmid-Borne β-Lactamase Genes in an Extended-Spectrum-β-Lactamase-Producing Escherichia coli Strain: Impact on Resistance and Proteomic Profile
Source: Microbiol Spectr. 2023 Jan 9;11(1):e03867-22. doi: 10.1128/spectrum.03867-22 (PMC9927464; doi:10.1128/spectrum.03867-22)
Supplement: Supplemental file 1 — Fig. S1 to S3, Tables S1 and S2, and Appendix S1. Download spectrum.03867-22-s0001.pdf, PDF file, 3.8 MB [file spectrum.03867-22-s0001.pdf]

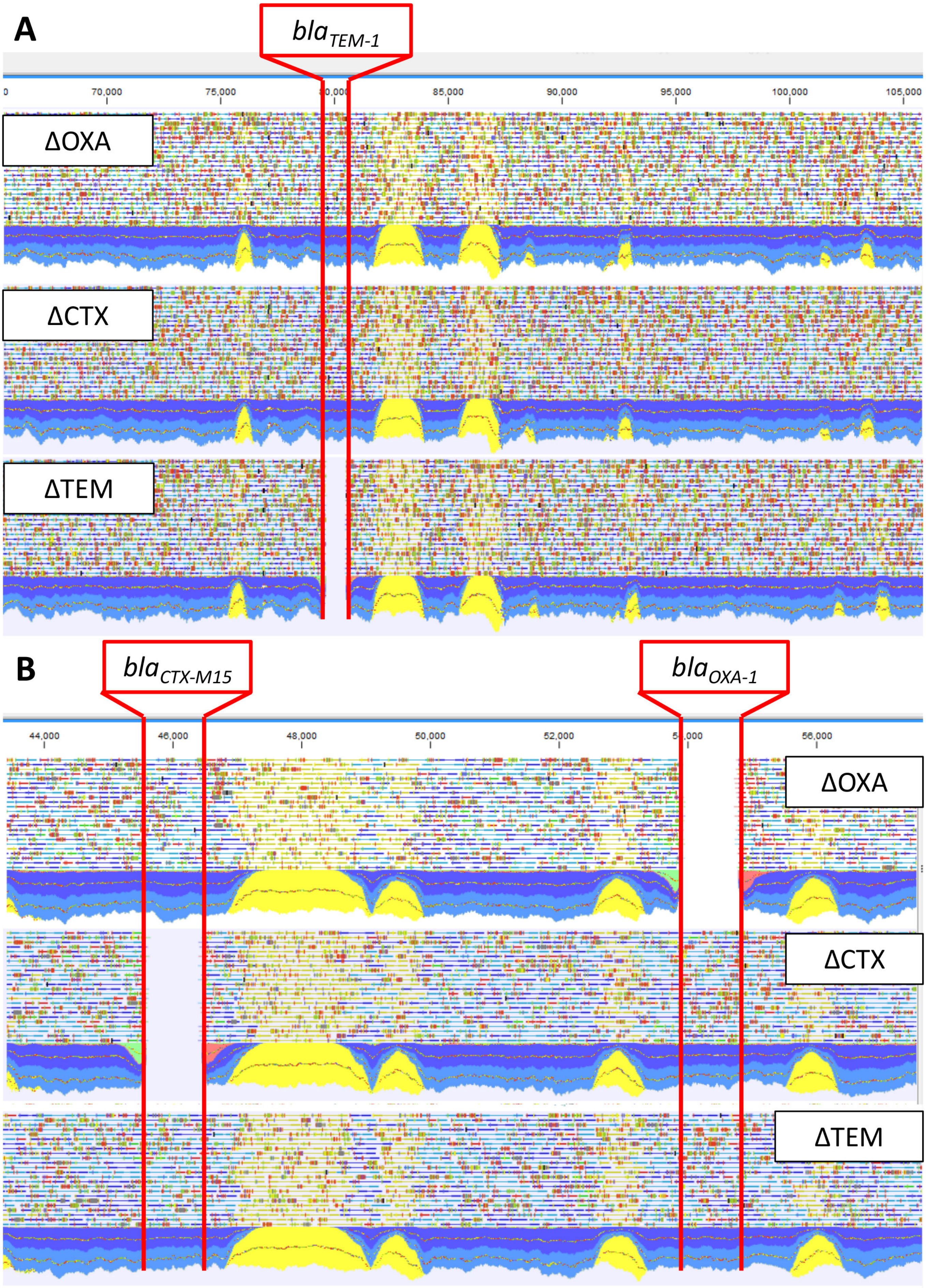

Fig. S1. Mapping of Illumina reads of the clones sequenced. The mapping was done over A) pSUH-1 (*bla*$_{TEM-1}$ encoding plasmid) and pSUH-2 (*bla*$_{CTX-M15}$ and *bla*$_{OXA-1}$ encoding plasmid) reference sequence of the Wild-Type strain *E. coli* CCUG 73778.

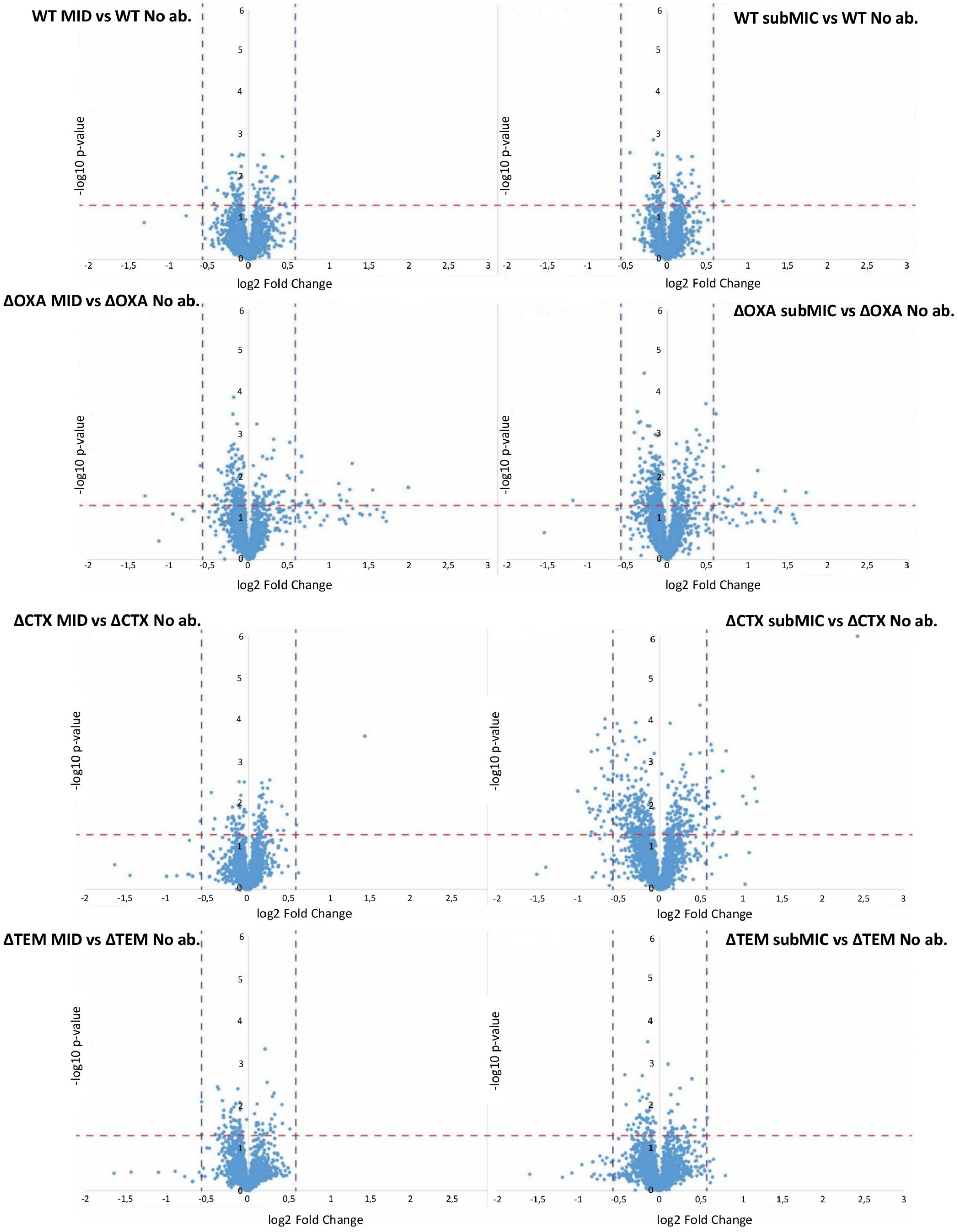

Fig. S2. Volcano plots of all four strains compared with the respective No antibiotic conditions in each case. WT and ΔTEM showed no significant changes in protein expression, ΔOXA a higher number of proteins which expression is significantly changed and ΔCTX shows the higher number of proteins surpassing the thresholds in subMIC conditions.

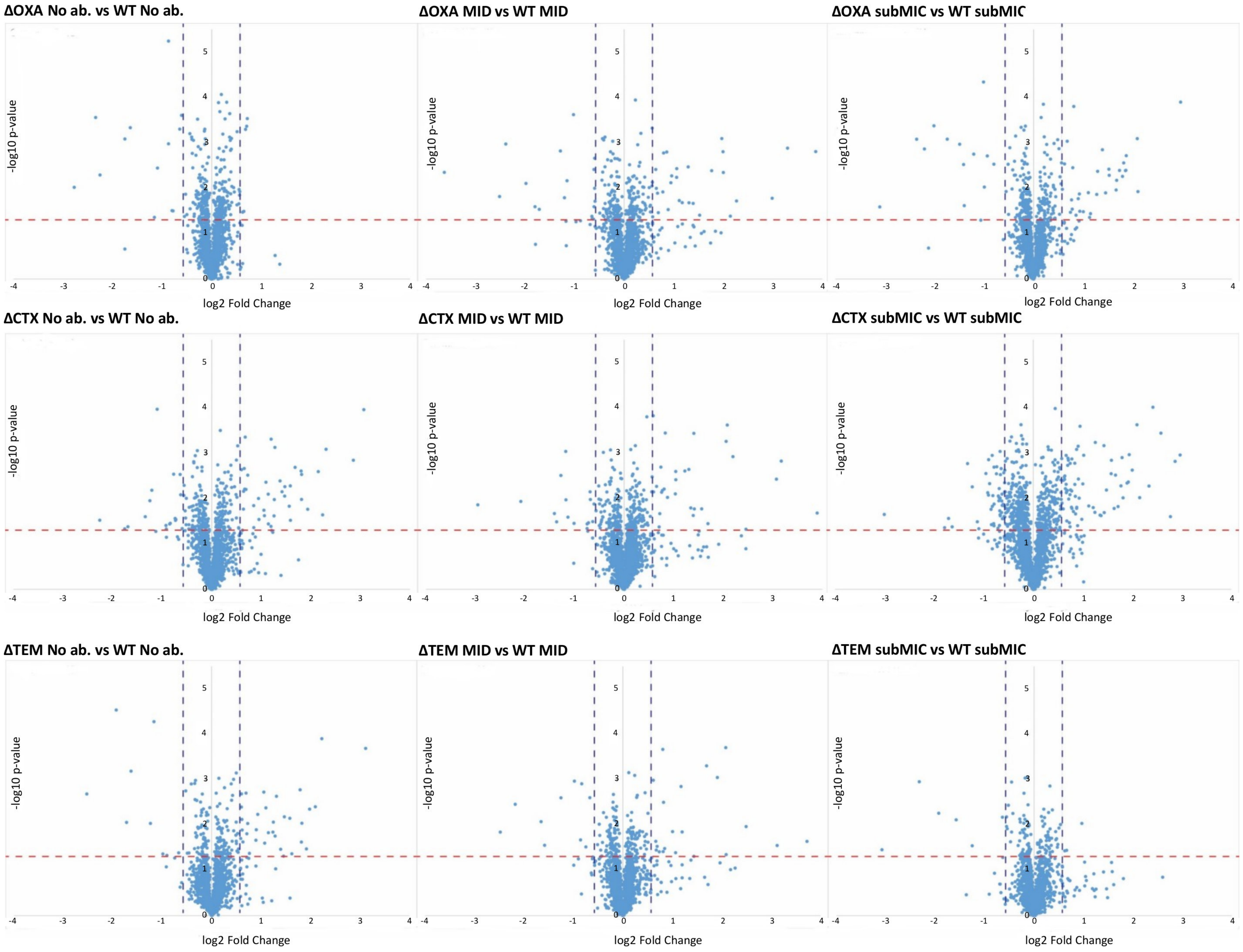

Fig. S3. Volcano plots of each clone variant compared with the WT in each condition. The overall change in expression of proteins from No antibiotic to subMIC conditions, with respect to the WT, can be seen.

Table S1. Primer sequences used in the present study.

Primer	Sequence	Target	Reference
TEMS1-F	AACGACGGCCAGTATAGGGATAACAGGGTAATCTGAATTCCTGCCGATATGATCCAACTGA	Region S1 upstream TEM-1	This work
TEMS1-R	CCAGGGTTATTGTATCATGAGC	Region S1 upstream TEM-1	This work
TEMS2-F	ACATTCAAATATGTATCCGCTCATGATACAATAACCCTGGACTGTCAGACCAAGTTTACTC	Region S2 downstream TEM-1	This work
TEMS2-R	CTAGAAGCTTGCATGCCTGCAGGTCGACTCTAGAGGATCCCGTCATCCGGTTGCGAATC	Region S2 downstream TEM-1	This work
OXAS1-F	AACGACGGCCAGTATAGGGATAACAGGGTAATCTGAATTCCGACACTTGCTGACGTACA	Region S1 upstream OXA-1	This work
OXAS1-R	TTAATGAGGCTCCGGGTTCG	Region S1 upstream OXA-1	This work
OXAS2-F	CCCTCATGTCAAACGTTGGGCGAACCCGGAGCCTCATTAACCAACCCTTCAATCAAGTCG	Region S2 downstream OXA-1	This work
OXAS2-R	CTAGAAGCTTGCATGCCTGCAGGTCGACTCTAGAGGATCCATTCCGGGCATGACCATTG	Region S2 downstream OXA-1	This work
CTXS1-F	AACGACGGCCAGTATAGGGATAACAGGGTAATCTGAATTCATGGCGGTGGGTCATCTCTTG	Region S1 upstream CTX-M15	This work
CTXS1-R	GGGATTCCTTATTCTGGAAGATACG	Region S1 upstream CTX-M15	This work
CTXS2-F	ATGTTGTTGTTATTTCGTATCTTCCAGAATAAGGAATCCCTAGCGGAAACGGAATGGGGAAAC	Region S2 downstream CTX-M15	This work
CTXS2-R	CTAGAAGCTTGCATGCCTGCAGGTCGACTCTAGAGGATCCGGACGCCGGAAGGAATATGAC	Region S2 downstream CTX-M15	This work
pEMG-F1	CCATTCAGGCTGCGCAACTGTTG	pSEVA312S	Martínez-García & de Lorenzo 2011
pEMG-R1	CTTTACACTTTATGCTTCCGGC	pSEVA312S	Martínez-García & de Lorenzo 2011
TEM-OUT-F	GTCAGGTAGGGGAACAACTGG	*E. coli* CCUG 73778, outside TEM-1 gene	This work
TEM-OUT-R	GTTAAGAAGCTCGACCGTCTTG	*E. coli* CCUG 73778, outside TEM-1 gene	This work
OXA-OUT-F	CTATGAAAAGCGTGGTAATGCTG	*E. coli* CCUG 73778, outside OXA-1 gene	This work
OXA-OUT-R	TATCACGAATCCCGAGCTGTCTG	*E. coli* CCUG 73778, outside OXA-1 gene	This work
CTX-OUT-F	TGCAACAGTGCCCCACATC	*E. coli* CCUG 73778, outside CTX-M15 gene	This work
CTX-OUT-R	GATGCTCTATGAGTGGCTAAATCG	*E. coli* CCUG 73778, outside CTX-M15 gene	This work
blaTEM-F	CGCCGCATACACTATTCTCAGAATGA	targets the original gene	Fang et al.
blaTEM-R	ACGCTCACCGGCTCCAGATTTAT	targets the original gene	Fang et al.
blaCTX-F	ATGTGCAGYACCAGTAARGTKATGGC	targets the original gene	Fang et al.
blaCTX-R	TGGGTRAARTARGTSACCAGAAYCAGCGG	targets the original gene	Fang et al.
blaOXA-F	ACACAATACATATCAACTTCGC	targets the original gene	Fang et al.
blaOXA-R	AGTGTGTTTAGAATGGTGATC	targets the original gene	Fang et al.

Table S2. Results of the disk diffusion test performed on *E. coli* CCUG 73778 (WT) and its knock-out clone variants. Diameter of the inhibition halo are indicated in millimeters (mm)

Antibiotic	WT (mm)	ΔOXA (mm)	ΔCTX (mm)	ΔTEM (mm)
Cefadroxil	0	0	15,7	0
Cefotaxime	0	0	25,5	0
Cephazolin	0	0	15	0
Cephalothin	0	0	12,8	0
Cefoxitin	22,8	23,5	24,1	22,5
Ceftazidime	12,6	12,8	26,4	11,5
Ceeftriaxome	7,5	7,8	30,2	7,3
Ceftibuten	19	19,5	28,5	18,3
Ertapenem	28,7	28,7	31,4	28,5
Tircacillin	0	0	0	0
Aztreonam	12	11,8	29	10,7
Piperacillin	0	0	8	0
Imipenem	28,8	30,2	30	28,5
Meropenem	30,6	30,8	32,3	31
Ciprofloxacin	30	30,7	29	30
Penicillin	0	0	0	0
Oxacillin	0	0	0	0
Cloxacillin	0	0	0	0
Ampicillin	0	0	0	0

S1 Appendix. Description of the content of the supplementary files.

Supplementary files are Excel files which contains results extracted from the proteomic information and the different comparisons performed. The information contained in each supplementary file is structured in different excel sheets.

S1 File. Proteins highlighted in Comparison 1. Fold changes and Welch's test p-values are indicated for each protein. Only proteins meeting the thresholds (Fold change (FC) >1.5. <-1.5; p-value <0.05), are indicated.

- Sheet 1. Comparison of WT in subMIC conditions over WT with no antibiotic.
- Sheet 2. Comparison of ΔOXA in MID conditions over ΔOXA with no antibiotic.
- Sheet 3. Comparison of ΔOXA in subMIC conditions over ΔOXA with no antibiotic.
- Sheet 4. Comparison of ΔCTX in MID conditions over ΔCTX with no antibiotic.
- Sheet 5. Comparison of ΔCTX in subMIC conditions over ΔCTX with no antibiotic.
- Sheet 6. Comparison of ΔTEM in subMIC conditions over ΔTEM with no antibiotic.

S2 File. Proteins highlighted in Comparison 2. Fold changes and Welch's test p-values are indicated for each protein. Only proteins meeting the thresholds (Fold change (FC) >1.5. <-1.5; p-value <0.05), are indicated.

- Sheet 1. Comparison of ΔOXA with no antibiotic over WT with no antibiotic.
- Sheet 2. Comparison of ΔOXA in MID concentration over WT in MID concentration.
- Sheet 3. Comparison of ΔOXA in subMIC concentration over WT in subMIC concentration.
- Sheet 4. Comparison of ΔCTX with no antibiotic over WT with no antibiotic.
- Sheet 5. Comparison of ΔCTX in MID concentration over WT in MID concentration.
- Sheet 6. Comparison of ΔCTX in subMIC concentration over WT in subMIC concentration.
- Sheet 7. Comparison of ΔTEM with no antibiotic over WT with no antibiotic.
- Sheet 8. Comparison of ΔTEM in MID concentration over WT in MID concentration.
- Sheet 9. Comparison of ΔTEM in subMIC concentration over WT in subMIC concentration.

S3 File. Proteins detected in subMIC conditions for each knock-out clone variant. For each protein, the fold-changes and statistics are presented from no exposure to antibiotic to maximum concentration (No Ab., MID and subMIC) when compared to the WT (Comparison 2). The table reflects the evolution of fold-changes and p-values for each protein highlighted at subMIC in the different conditions used.

- Sheet 1 Trends of proteins highlighted in ΔOXA in Comparison 2.
- Sheet 2. Trends of proteins highlighted in ΔCTX in Comparison 2.
- Sheet 3. Trends of proteins highlighted in ΔTEM in Comparison 2.
